# A novel peptide PDHK1-241aa encoded by circPDHK1 promotes ccRCC progression via interacting with PPP1CA to inhibit AKT dephosphorylation and activate the AKT-mTOR signaling pathway

**DOI:** 10.1186/s12943-024-01940-0

**Published:** 2024-02-15

**Authors:** Bo Huang, Junwu Ren, Qiang Ma, Feifei Yang, Xiaojuan Pan, Yuying Zhang, Yuying Liu, Cong Wang, Dawei Zhang, Ling Wei, Lingyu Ran, Hongwen Zhao, Ce Liang, Xiaolin Wang, Shiming Wang, Haiping Li, Hao Ning, Ai Ran, Wei Li, Yongquan Wang, Bin Xiao

**Affiliations:** 1 College of Pharmacy, Chongqing Medical University, Chongqing, 400016 P.R. China; 2 Key Laboratory of Basic Pharmacology of Ministry of Education and Joint International Research Laboratory of Ethnomedicine of Ministry of Education, Zunyi Medical University, Zunyi, Guizhou 563006 P.R. China; 3 Department of Urology, Southwest Hospital, Army Medical University, Chongqing, 400038 P.R. China; 4 Department of Kidney, Southwest Hospital, Army Medical University, Chongqing, 400038 P.R. China; 5 Department of Pharmacy, Chongqing University Cancer Hospital, Chongqing, 400030 P.R. China

**Keywords:** Clear cell renal cell carcinoma, CircPDHK1, Novel peptide, PPP1CA, Dephosphorylation

## Abstract

**Background:**

Clear cell renal cell carcinoma (ccRCC) is the most prevalent kidney cancer with high aggressive phenotype and poor prognosis. Accumulating evidence suggests that circRNAs have been identified as pivotal mediators in cancers. However, the role of circRNAs in ccRCC progression remains elusive.

**Methods:**

The differentially expressed circRNAs in 4 paired human ccRCC and adjacent noncancerous tissues ccRCC were screened using circRNA microarrays and the candidate target was selected based on circRNA expression level using weighted gene correlation network analysis (WGCNA) and the gene expression omnibus (GEO) database. CircPDHK1 expression in ccRCC and adjacent noncancerous tissues (*n* = 148) were evaluated along with clinically relevant information. RT-qPCR, RNase R digestion, and actinomycin D (ActD) stability test were conducted to identify the characteristics of circPDHK1. The subcellular distribution of circPDHK1 was analyzed by subcellular fractionation assay and fluorescence in situ hybridization (FISH). Immunoprecipitation-mass spectrometry (IP-MS) and immunofluorescence (IF) were employed to evaluate the protein-coding ability of circPDHK1. ccRCC cells were transfected with siRNAs, plasmids or lentivirus approach, and cell proliferation, migration and invasion, as well as tumorigenesis and metastasis in nude mice were assessed to clarify the functional roles of circPDHK1 and its encoded peptide PDHK1-241aa. RNA-sequencing, western blot analysis, immunoprecipitation (IP) and chromatin immunoprecipitation (ChIP) assays were further employed to identify the underlying mechanisms regulated by PDHK1-241aa.

**Results:**

CircPDHK1 was upregulated in ccRCC tissues and closely related to WHO/ISUP stage, T stage, distant metastasis, VHL mutation and Ki-67 levels. CircPDHK1 had a functional internal ribosome entry site (IRES) and encoded a novel peptide PDHK1-241aa. Functionally, we confirmed that PDHK1-241aa and not the circPDHK1 promoted the proliferation, migration and invasion of ccRCC. Mechanistically, circPDHK1 was activated by HIF-2A at the transcriptional level. PDHK1-241aa was upregulated and interacted with PPP1CA, causing the relocation of PPP1CA to the nucleus. This thereby inhibited AKT dephosphorylation and activated the AKT-mTOR signaling pathway.

**Conclusions:**

Our data indicated that circPDHK1-encoded PDHK1-241aa promotes ccRCC progression by interacting with PPP1CA to inhibit AKT dephosphorylation. This study provides novel insights into the multiplicity of circRNAs and highlights the potential use of circPDHK1 or PDHK1-241aa as a therapeutic target for ccRCC.

**Supplementary Information:**

The online version contains supplementary material available at 10.1186/s12943-024-01940-0.

## Introduction

Clear cell renal cell carcinoma (ccRCC) is the most prevalent subtype of kidney cancer, accounting for more than 75% of all renal cell carcinoma (RCC) cases. Histologically, ccRCC is characterized by the appearance of thin-walled cells filled with abundant lipids and glycogen [1, 2]. Notably, if ccRCC tumors exceed 7 cm in size or have metastasized, the 5-year survival rate is less than 10%. Although significant advancements have been made in the treatment of ccRCC through the development of targeted drugs and immunotherapy, many patients with advanced ccRCC do not exhibit favorable responses to these therapeutic approaches, resulting in poor long-term prognosis [3–5]. The pathogenesis of ccRCC is unclear, although it may be related to genetic and chromosomal abnormalities, including gene fusion [1, 3]. Therefore, further mechanistic dissection of ccRCC growth and metastasis is imperative to develop innovative therapeutic strategies.

Circular RNAs (circRNAs) are a subtype of noncoding RNAs characterized by covalently closed, single-stranded transcripts lacking 5’ to 3’ polarity or poly A tails. These circRNAs exist in various eukaryotes and exhibit higher stability than their linear counterpart mRNAs [6]. Owing to their abundance and stability, circRNAs regulate a wide range of biological activities. They function as competing endogenous RNAs (ceRNAs), interacting with RNA-binding proteins or modulating transcription and splicing [7–9]. CircRNAs have also been implicated in the tumorigenesis and development of ccRCC. For instance, circCHST15 regulates the miR-125-5p/EIF4EBP1 axis to promote ccRCC progression [10]. Our previous research demonstrated that circTNPO3 suppresses ccRCC proliferation and migration by interacting with IGF2BP2 [11]. Furthermore, emerging evidence suggests that circRNAs, despite being classified as noncoding RNAs, can generate functional peptides or proteins. The expression of open reading frames (ORFs) in circRNAs is facilitated by internal ribosomal entry site (IRES) or N6-methyladenosine (m6A) modifications. CircEIF6, circMAP3K4, and circNSUN2 have been identified as translatable circRNAs that play important roles in cancer development [12–14]. However, it remains unclear whether protein coding circRNAs are involved in ccRCC tumorigenesis, and functional protein products have not yet been identified.

The majority of ccRCC cases exhibit inactivation or mutation of the Von Hippel‒Lindau (VHL) gene [15–17]. VHL is a substrate-recognition component of the E3 ligase complex. VHL loss leads to aberrant accumulation of hypoxia-inducible factor (HIF) proteins by regulating the ubiquitin‒proteasome system. HIF-1A and HIF-2A are the two most important HIF subtypes [18, 19] that play distinct roles in ccRCC. HIF-2A plays a crucial oncogenic role in ccRCC, whereas HIF-1A appears to be a tumor suppressor [20]. The oncogenic effects of HIF-2A in ccRCC are multifaceted, with a key role in enhancing the transcriptional activity of target genes [21, 22].

The phosphoprotein phosphatase (PPP) family members include PP1, PP2A (PP2), PP2B (PP3), and PP4 [23]. PP1 and PP2A, as the major eukaryotic protein phosphatases in the PPP family that are reported to participate in over 90% of serine/threonine dephosphorylation and regulate various cellular processes through the dephosphorylation of different substrates [24]. Protein phosphatase 1 (PP1) is a member of the major serine/threonine-specific protein phosphatase family that is responsible for dephosphorylating its target proteins and participating in the regulation of various cellular processes [25]. PP1 consists of three catalytic subunits, PPP1CA, PPP1CB, and PPP1CC. Notably, PPP1CA played a significant role in several cellular pathways. For instance, PPP1CA dephosphorylates B-Raf at both ERK phosphorylation sites to trigger PPP1CA/B-Raf/ERK pathway activation and promote prostate cancer (CaP) cell invasiveness [26]. Furthermore, PPP1CA directly dephosphorylates AKT, thereby controlling gene expression, cell survival, and differentiation [27, 28].

In the present study, we identified an upregulated circRNA in ccRCC, circPDHK1 (hsa_circ_0057090), and its expression was positively correlated with tumor grade in patients with ccRCC. It has been reported that PDHK1 encoded by linearPDHK1 promotes the growth and metastasis of cancer by regulating the aerobic glycolysis of tumor cells [29, 30]. Through in vitro and in vivo functional investigations, we demonstrated that circPDHK1 enhanced the proliferation and metastatic ability of cultured ccRCC cells. Additionally, we revealed that circPDHK1 encodes a novel peptide, PDHK1-241aa, which is responsible for the oncogenic function of circPDHK1. Mechanistically, PDHK1-241aa interacts with PPP1CA, resulting in the nuclear translocation of PPP1CA. This process leads to reduced dephosphorylation of AKT and subsequent activation of the AKT-mTOR signaling pathway, thereby promoting ccRCC proliferation and metastasis.

## Materials and methods

### Patients and tissue samples

We collected 152 paired human ccRCC and adjacent nontumor tissues (4 paired tissues for screening phase and 148 paired tissues for validation phase) from the Southwest Hospital of the Army Medical University (Chongqing, China, 2019–2022). Clinical characteristics are listed in Supplementary Table S1-2. The diagnosis of ccRCC was confirmed via histopathological examination. This study was approved by the Ethics Committee of Southwest Hospital of Army Medical University (approval number: KY2020121). Written informed consent was obtained from each participant prior to the surgery. The tissues were immediately preserved in RNAlater (Thermo Fisher, Shanghai, China) for storage.

### Microarray analysis

We performed microarray to identify differentially expressed circRNAs in 4 pairs of ccRCC and adjacent noncancerous tissues. RNA extraction, quality identification, analysis of differentially expressed circRNAs were performed by Shanghai Genomics Corporation (Shanghai, China) as described previously [11].

### Cell lines and cell culture

Caki-1, 786-O, RCC-JF, RCC-23 (ccRCC cell lines), HK-2 (adult renal tubular epithelial cells) and 293 T (normal human embryonic kidney cell lines) were purchased from Meisen CTCC (Zhejiang, China). All cell lines were authenticated via STR profiling. 293 T and HK-2 cells were cultured in DMEM (HyClone, Logan, Utah, USA), Caki-1 cells were cultured in McCoy’s 5A (Basal Media, Shanghai, China), and 786-O, RCC-23 and RCC-JF cells were cultured in RPMI-1640 (HyClone). All media were supplemented with 10% FBS (PAN, Aidenbach, Germany), 1% penicillin/streptomycin (Biosharp, China) and 1‰ Mycoplasma Elimination Reagent (Yeasen, Shanghai, China) to remove mycoplasma. The cells were cultured in 5% CO_2_ at 37 °C.

### Actinomycin D (ActD) assay

Caki-1 and 786-O cells were seeded into 12-well plates the day before the experiment. cells were treated with 10 μg / mL actinomycin D (Genview, Beijing, China) for 0, 6, 12 or 24 h to inhibit RNA transcription. Total RNA was extracted from Caki-1 and 786-O cells using TRIzol reagent (Takara, Shiga, Japan) according to the manufacturer’s protocol. Followed by reverse transcription using PrimeScript RT (Takara, Shiga, Japan). The cDNA was amplified by qPCR using 2 × SP qPCR Mix (BioGround, China) on a CFX96 Real-Time PCR System (BioRad, USA). GAPDH were used as internal references and calculated using the 2^−ΔΔCt^ method.

### RNase R treatment

Total RNA was extracted from Caki-1 and 786-O cells using TRIzol reagent (Takara, Shiga, Japan) according to the manufacturer’s protocol. 5 μg RNA of Caki-1 and 786-O cells were treated with RNase R (6 U) (Lucigen, Middleton, WI, USA) at 37 °C for 10 min and 85 °C for 5 s according to the manufacturer’s instructions. For controls, 5 µg of total RNA was mock treated under the same conditions without the enzyme. The treated RNAs were subjected to reverse transcription using PrimeScript RT reagent Kit (Takara, Shiga, Japan) following the manufacturer’s instructions. The primers for circPDHK1 and linear PDHK1 were synthesized by Tsingke (Tsingke, China). The expression levels of circPDHK1 and linear PDHK1 were assessed using RT-qPCR with the Bio-Rad CFX96™ Real-Time PCR System (BioRad, USA).

### Plasmids, siRNAs and cell transfection

The circPDHK1 siRNA (si-circPDHK1#1) and PPP1CA siRNA (si-PPP1CA) utilized in the experimental procedures were synthesized by GenePharma (Shanghai, China). Additionally, circPDHK1 siRNA (si-circPDHK1#2) was synthesized by Tsingke (Beijing, China). The specific siRNA sequences are listed in Supplementary Table S3. The IRES and its truncated mutants were synthesized and cloned and inserted into the Luc2-Report plasmid (Geneseed, Guangdong, China) using *EcoRI* and *BamHI* sites. Full-length circPDHK1 and circPDHK1-3 × flag was synthesized and cloned and inserted into the pLC5-ciR vector (Geneseed) using *EcoRI* and *BglII* sites. The 3 × flag sequence is located after the start codon ATG in the predicted coding sequence (CDS) of circPDHK1. The circPDHK1-3 × flag ATG mutation overexpression vector was constructed by QuickMutation™ Site-Directed Mutagenesis Kit (Beyotime, Shanghai, China) following the manufacturer’s instructions. The human PPP1CA overexpression vector pCDH-PPP1CA was synthesized by Tsingke, whereas the human HIF-2A overexpression vector pCDNA3.1-HIF-2A was obtained from Unibio (Chongqing, China). The plasmids were transfected using a commercial DNA transfection reagent (Neofect, Beijing, China) following the manufacturer’s instructions. siRNAs were transfected using Lipo8000 transfection reagent (Beyotime, Shanghai, China) following the manufacturer’s instructions.

### Cell counting kit-8 assay

Caki-1 and 786-O cells were collected and seeded into 96-well plates at a density of 3000 cells per well. Subsequently, 90 µL complete medium and 10 µL CCK-8 solution (TargetMol, USA) was added to each well at 0 h, 24 h, 48 h, 72 h, 96 h. The timing was started after the cells were attached to the well. The cells were incubated at 37 °C for 2 h. The absorbance at 450 nm was measured with Multiskan SkyHigh microplate reader (Thermo, China). The obtained data were analyzed using GraphPad 7.0 software (GraphPad, San Diego, CA, USA).

### Colony formation assay

Caki-1 and 786-O cells (3 × 10^3^ cells per well) were seeded in 12-well plates to assess their proliferative ability and incubated in complete medium for two weeks. Then, the cells were washed with PBS, fixed with 4% paraformaldehyde solution (Biosharp, China), and stained with 1% crystal violet staining solution (Beyotime, Shanghai, China), and the images were obtained using a scanner (EPSON, Japan).

### 5-ethynyl-2’-deoxyuridine (EdU) assay

EdU assays were used to assess cell proliferation capacity using the Click-iT EdU kit (Bioscience, Shanghai, China). Briefly, 2 × 10^4^ ccRCC cells were seeded into 48-well plates and incubated for 16 h, followed by incubation with 25 µM EdU reagent at 37 °C for 2 h. The cells were fixed with 4% paraformaldehyde solution (Biosharp, China) for 15 min. After fixation, cells were incubated with 0.5% Triton X-100 for 10 min, stained with YF®594Azide Click-iT EdU for 30 min in the dark, and incubated with Hoechst 33,342 for 15 min. Cells were visualized by fluorescence microscopy (Lecai, Germany). The proliferation rate of cells was determined by calculating the ratio of EdU-positive nuclei to total nuclei.

### Migration and invasion assay

Transwell plates (Corning, NY, USA) were used for the migration and invasion assays. Briefly, for transwell migration assay, 3 × 10^4^ cells were plated in medium without serum in the upper chamber of a transwell with an 8.0 μm pore size. The lower chamber was filled with 500 µL complete medium. Likewise, for invasion assay, prepare Matrigel working solution proportionally (Matrigel: serum-free medium = 1:8) was added to the upper chamber and incubated at 37 °C for 2 h. Then, 6 × 10^5^ cells were seeded in Matrigel-coated chambers with serum-free medium. After incubation for 24 h, the Transwell filters were fixed with 4% paraformaldehyde solution (Biosharp, China), stained with 1% crystal violet staining solution (Beyotime, Shanghai, China), and photographed by same microscopy. The number of migrated cells was counted using ImageJ software. Additionally, a wound healing assay was performed to detect ccRCC migration following a previously described protocol [11, 31]. ccRCC cells transfected in 6-well plates were scratched 48 h after transfection. The medium need replaced with serum-free medium the night before the wound healing assay. Then, a sterile 10 µL plastic pipette tip (Labselect, China) was used to scratch through the single-cell layer, and images were captured after 0 h, 12 h and 24 h at the same place with a microscope (Nexscope, China). The cell migration distances were counted using ImageJ software.

### Western blot

Patient tissues or cultured cells were lysed using RIPA buffer supplemented with a protease inhibitor cocktail and 1% PMSF. To isolate cytoplasmic and nuclear proteins, we used a Nuclear and Cytoplasmic Protein Extraction Kit (Beyotime, Shanghai, China) following the manufacturer’s instructions. Protein samples were separated by sodium dodecyl sulfate‒polyacrylamide gel electrophoresis (SDS‒PAGE) and subsequently transferred to polyvinylidene fluoride (PVDF) membranes (0.2 μm) by electroblotting. After they were blocked in 5% nonfat milk for 1 h, the membranes were incubated overnight at 4 ℃ with the indicated primary antibodies. Next, they were incubated with horseradish peroxidase (HRP)-conjugated secondary antibodies for 1 h at room temperature and visualized with the enhanced chemiluminescence (ECL) detection reagent (Bioscience, China). The antibodies used in the present study were as follows: rabbit anti-PDHK1/PDHK1-241aa (Novus, Centennial, CO, USA, NBP1-85955, 1:1000 dilution), rabbit anti-Flag (CST; Cell Signaling Technology, Danvers, MA, USA, #14793S, 1:1000 dilution), mouse anti-Flag (Beyotime, AF519-1, 1:1000 dilution), rabbit anti-PPP1CA (ABclonal, Wuhan, China, A12468, 1:1000 dilution), rabbit anti-HIF-2A (CST, #59973S, 1:1000 dilution), rabbit anti-pan-AKT (CST, #4691S, 1:1000 dilution), rabbit anti-phospho-AKT Thr308 (CST, #13038S, 1:1000 dilution), rabbit anti-mTOR (CST, #2983S, 1:1000 dilution), rabbit anti-phospho-mTOR Ser2448 (CST, #5536S, 1:1000 dilution), rabbit anti-β-actin (ZSGB-BIO, China, ZM-0001, 1:1000 dilution), mouse anti-GAPDH(Beyotime, AF0006, 1:1000 dilution), rabbit anti-α-tubulin (Beyotime, AF5012, 1:1000 dilution), mouse anti-histone H3 (Beyotime, AF0009, 1:1000 dilution), rabbit anti-PI3K (CST, Cell Signaling Technology, Danvers, MA, USA #4257S, 1:1000 dilution), rabbit anti-p-PI3K (CST; Cell Signaling Technology, Danvers, MA, USA, #4288 T, 1:1000 dilution), rabbit anti-PTEN (CST; Cell Signaling Technology, Danvers, MA, USA, #9559 T, 1:1000 dilution), rabbit anti-PDK1 (Proteintech, Wuhan, China, 17086–1-AP, 1:1000 dilution).

### Immunofluorescence (IF) assay

IF assays were employed to investigate the subcellular localization of PDHK-241aa and PPP1CA following previously described methods [32]. Briefly, the transfected cells were incubated in u-Slide 8 well plates (Ibidi, Germany) with 2 × 10^4^ cells per well one day in advance. The cells were fixed with 4% paraformaldehyde solution (Biosharp, China) for 15 min. After fixation, the cells were incubated with 0.5% Triton X-100 for 10 min at room temperature. Cells were incubated with 10% goat serum for 60 min at room temperature and incubated with the primary antibody (1:200 dilution) at 4 ℃ for overnight. After incubation with secondary antibody (1:100 dilution) combined with fluorescent dye for 1 h in the dark. Next, the cells were incubated with DAPI for 10 min. Images of the slides were obtained using a fluorescence microscope (Leica SP8, Wetzlar, Germany).

### Immunohistochemistry (IHC) assay

IHC assays of mouse xenograft tumor tissues were performed by Servicebio (Wuhan, China). Proliferative markers were detected using anti-Ki-67 (Bioss, Beijing, China). Images were obtained using a fluorescence microscopy (Lecai, Germany).

### RNA fluorescence in situ hybridization (FISH) assay

FISH assays were employed to determine the subcellular localization of circPDHK1 following previously described protocols [11]. Briefly, the Cy3-labeled circPDHK1 probe and FISH probe kit were synthesized by GenePharma (Shanghai, China. Supplementary Table S4). The cells were seeded in u-Slide 8 well plates (Ibidi, Germany) at a density of 2 × 10^4^ cells/well. Cells were grown to 50% confluence and then fixed with 4% paraformaldehyde. The hybridization experiments were performed using a fluorescence in situ hybridization kit (GenePharma, China) according to the manufacturer’s protocol. 18 s ribosomal RNAs (rRNAs) were used as positive controls. Then, cell nuclei were stained with DAPI and observed under a fluorescence confocal microscope (Leica SP8, Wetzlar, Germany).

### Co‒immunoprecipitation (Co‒IP) assay

A Pierce Crosslink Magnetic IP/Co-IP Kit (Thermo Fisher, Shanghai, China) was used for immunoprecipitation experiments. Briefly, 1 × 10^7^ cells were lysed with 1 mL Pierce™ IP lysis buffer (Thermo Scientific, USA) for 20 min on ice. Then, the supernatants were collected after centrifugation (13,000 g × 15 min). 25 μL protein A/G magnetic beads and antibodies or control IgG (5 µg antibody) were added to the centrifuge tubes and incubated 15 min at room temperature on a rotator. Then, using antibody-crosslinked magnetic beads by 20 µM Dextran sulfate sodium (DSS) at room temperature for 45 min on a rotator. The lysates were incubated with magnetic beads at 4 °C overnight on a rotator. 100 µL eluent buffer was added to retain the supernatant containing the target antigen for 5 min on a rotator, which was then neutralized and subjected to SDS‒PAGE and mass spectrometry (MS) analysis.

### Liquid chromatography-mass spectrometry (LC–MS) analysis

The LC–MS analysis was performed by Sangon Biotech (Shanghai, China). Briefly, proteins were separated using SDS‒PAGE, followed by overnight trypsin digestion and subsequent desalting. The digested peptides were analyzed using a Q Exactive Plus LC–MS instrument (Thermo Fisher, Pittsburg, PA, USA), and the spectra were analyzed using Protein Pilot Ver. 4.5 supplied by the instrument.

### RNA/gDNA extraction, RT-PCR and RT-qPCR assays

Total RNA was extracted from cell lines and tissues using TRIzol reagent (Takara Bio, Shiga, Japan) according to the manufacturer’s protocol. In brief, tissues or cells were lysed in 1 ml of TRIzol reagent. Subsequently, 200 µl chloroform was added to the lysis buffer for 10 min, followed by centrifugation for 15 min at 13,000 g, 4 °C. After centrifugation, the aqueous phase containing the RNA was transferred to a new tube and isopropanol was added to the aqueous phase at an equal volume for 10 min at 4 °C, followed by centrifugation at 13,000 g for 10 min at 4 °C. Next, the pellet containing the RNA was retained, and 1 ml of 75% ethanol was added to the tube, followed by centrifugation at 13,000 g for 10 min at 4 °C. The pellet was retained and mixed with 30–50 µL RNase-free water. Then, the RNA was stored at − 80℃ until further use. The genomic DNA (gDNA) was extracted using the Universal Genomic DNA Kit (CWBIO, China) according to the manufacturer’s protocol. For analysis of circRNA or mRNA, cDNA was randomly or oligo(dT) primed from 500 ng of total RNA using PrimeScript RT reagent Kit (Takara, Shiga, Japan) following the manufacturer’s instructions. Real-time qPCR was performed using commercial 2 × SP qPCR Mix (Bioground, China). GAPDH or U6 was used as an endogenous control. The relative expression levels of target genes were calculated following the formula of 2^−ΔΔCt^. All primers were synthesized by Tsingke (Beijing, China) and are listed in Supplementary Table S5.

### RNA-seq analysis

To determine downstream targets and pathways of circPDHK1, total RNA extracted from Caki-1 cells transfected with circPDHK1 siRNA, circPDHK1 overexpression vector, circPDHK1 ATG mutant vector or control siRNA/vector. Then, RNA-seq and KEGG analysis were performed by Shanghai Genomics Corporation (Shanghai, China) including library construction and computational analysis.

### Subcellular fractionation assay

A PARIS™ Kit (Invitrogen, USA) was used for the subcellular fractionation assay. 5 × 10^6^ cells were lysed with 300 µL cell separation buffer and incubated for 10 min on ice. The supernatant (cytoplasmic part) was added into RNase-free EP tube, and the precipitate (nuclear part) was added with 300 µL cell disruption buffer on ice. Then, the same amount of 2 × Lysis/Binding Solution and absolute ethanol were added to both the nuclear and cytoplasmic parts. After mixing, the mixture was added to a centrifuge column, centrifuged at 10,000 g for 1 min with the liquid was discarded. Finally, the eluate was added to eluting the RNA. The extracted RNA was subjected to reverse transcription and RT-qPCR to detect the expression of circRNA in the nucleus and cytoplasm. The U6 internal control was tested as follows: The expression of U6 was detected using the hairpin it™ U6 snRNA Quantitative detection kit (GenePharma, China). RNA reverse transcription system was as follows: 4 μL 5 × MMLV RT Buffer, 0.75 μL dNTP, 1.2 μL U6 snRNA RT primer mix, 0.2 μL MMLV Reverse Transcriptase, 1–3 μg RNA, RNase Free H_2_O To 20 μL. The reverse transcription reaction procedure was as follows: 25℃ for 30 min, 42℃ for 30 min, 85℃ for 5 min, 4℃. RT-qPCR was performed using the following system: 5 μL 2 × real-time PCR mix, 0.2 μL MiRNA/U6 snRNA specific primer, 0.1 μL Taq DNA polymerase, 1 μL RT product, RNase-Free H_2_O To 10 μL. The reaction procedure was as follows: 95°C for 3 min, (40 cycles of the following steps) 95℃ for 12 s, 62℃ for 40 s. The relative subcellular localization of circPDHK1 were calculated following the formula of 2^−ΔΔCt^.

### Dual‑luciferase reporter assay

For IRES activity analysis, 293 T cells were transfected with the IRES and its truncated mutant vectors and Renilla expression plasmids per well using Neofect DNA transfection reagent in 96-well plates. After 24 h of transfection, the cells were lysed with passive lysis buffer (Promega, USA), and Firefly and Renilla luciferase activities were measured using the Dual-Glo luciferase assay kit (Promega, Madison, WI, USA). To verify whether HIF-2A binds to the PDHK1 promoter region. The sequence of PDHK1 promoter was subcloned into the Luc2-report Plamsid using *BamHI* and *EcoRI* sites. Caki-1 and 786-O cells were transfected with the Luc2-PDHK1 promoter vectors and Renilla expression plasmids per well using Neofect DNA transfection reagent in 96-well plates. Firefly and Renilla luciferase activities were measured using the Dual-Glo luciferase assay kit (Promega, Madison, WI, USA). The fluorescence intensity was determined using a fluorescence microplate reader (Thermo, USA). The ratio of Firefly/Renilla luminescence was calculated to determine the relative luciferase activity.

### Chromatin immunoprecipitation(ChIP) assay

To investigate the interaction between HIF-2A and the promoter region of PDHK1, ChIP assays were performed using a Magnetic ChIP Kit (Merck, Darmstadt, Germany) according to the manufacturer’s procedure in our study. Briefly, Caki-1 and 786-O cells were incubated with 1% formaldehyde and neutralized with glycine solution. DNA fragments within the range of 200–1000 bp were obtained by ultrasonication. The nuclear lysate was immunoprecipitated using anti-HIF-2A or anti-IgG antibodies. Transcription factor-binding sites were predicted using Contra Ver. 3 http://bioit2.irc.ugent.be/contra/v3. The purified DNA fragments were further analyzed by RT-qPCR using specific primers (Supplementary Table S5).

### RNA immunoprecipitation (RIP) assay

RIP experiments were conducted using the Magna RNA-binding protein immunoprecipitation kit (Millipore, Burlington, MA, USA). The cells were processed at 1 × 10^7^ cells/reaction density and lysed by adding lysis buffer containing protease and RNase inhibitors for 5 min. The magnetic beads were combined with 5 μg antibody and control IgG at room temperature, and the corresponding cell lysates were added; the samples were incubated overnight at 4 °C. The samples were subjected subsequently to RNA purification and protein extraction. The immunoprecipitated RNA obtained from Caki-1 cells was validated by RT-qPCR with specific primers (Supplementary Table S5).

### In vivo tumorigenesis and metastasis assays

786-O and Caki-1 subcutaneous tumor-bearing male NSG mice were purchased from the Shanghai Model Organisms Center and randomly assigned to groups based on tumor size. In the siRNA xenograft tumor model group (*n* = 6, each group), cholesterol-modified circPDHK1-siRNA (10 nmol, GenePharma) was intratumorally injected once every two days for five consecutive weeks. Tumor volumes were measured every 2 d. Tumor tissues were excised and weighed after mice were euthanized, fixed, and stained for immunohistochemistry. In the xenograft tumor model overexpression group (*n* = 5, each group), a lentiviral vector containing cloned inserts (Oligobio, Beijing) was injected intratumorally for 18 days. Tumor volume measurements and processing were carried out as described above. For the lung metastasis model, stably transfected Caki-1 cells (6 × 10^6^ cells/0.2 mL PBS) were injected into the tail vein of 4-week-old male mice. After 48 days, the mice were assessed using the Aura Spectral Instruments in vivo Imaging System and sacrificed. Lung tissues were excised, photographed, and stained with hematoxylin and eosin (H&E), and the lung metastatic nodes were observed. All the animal experiments were conducted according to the protocol approved by the Ethics Committee of Chongqing Medical University (approval number: 2022013).

### Statistical analysis

One-way analysis of variance, Student’s t test, and chi-squared test were used to analyze the means between different groups. Statistical differences were calculated using Prism 7.0 (GraphPad, San Diego, CA, USA). The results are presented as the mean ± standard deviation (SD). In silico expression analyses were conducted with a cutoff of │log_2_FC│ ≥ 1 and weighted gene correlation network analysis (WGCNA) using per kilobase of exon per million mapped fragments (FPKM) > 1 and expressed in at least three samples. Statistical significance was set at *P* < 0.05.

## Results

### Screening and identification of upregulated circPDHK1 expression in ccRCC tissues and cells

**Fig. 1:**
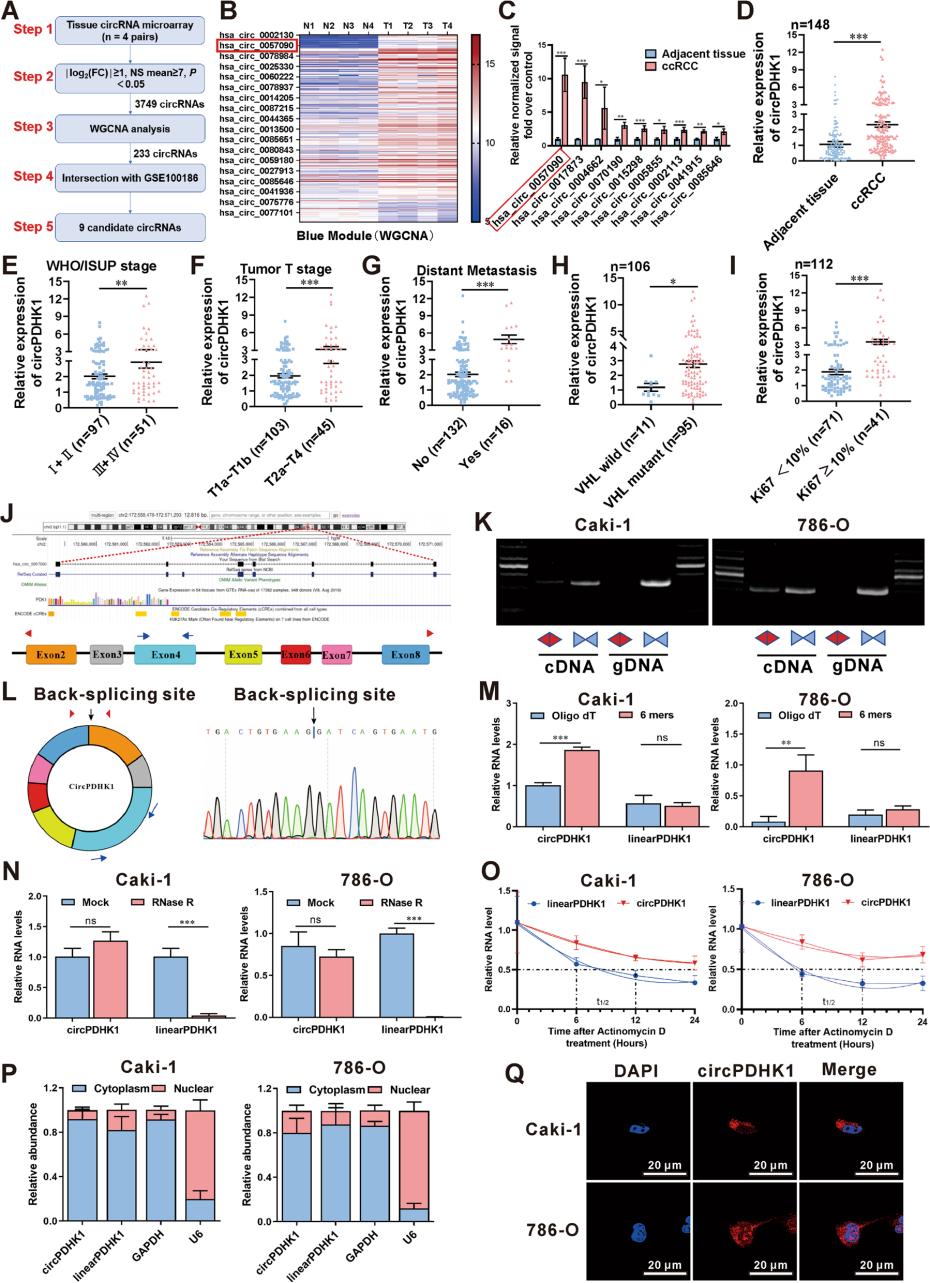
Identification and characteristics of upregulated circPDHK1 in ccRCC. **A** The workflow for circRNA screening in 4 paired ccRCC tissues as indicated. **B** Cluster heatmap showed the differentially expressed 233 circRNAs in the ME blue module with WGCNA generated from 4 ccRCC tissues and paired adjacent normal tissues. The red and blue strips indicate up-regulated and down-regulated circRNAs, respectively. **C** Relative normalized signal detected by circRNA probe in microarray analysis of the 9 candidate circRNAs that intersected with the 233 circRNAs in the GSE100186 data. **D** RT-qPCR analysis of circPDHK1 expression in 148 paired ccRCC samples and normal adjacent tissues. **E** Comparison of circPDHK1 expression between patients with WHO/ISUP stage III–IV (*n* = 51) and those with WHO/ISUP stage I–II (*n* = 97), detected by RT-qPCR. **F** Comparison of circPDHK1 expression between patients with tumor T stage T1a-T1b (*n* = 103) and those with tumor T stage T2a-T4 (*n* = 45), detected by RT-qPCR. **G** Association analyses of circPDHK1 expression and distant tumor metastasis. **H** Relationships between circPDHK1 expression and VHL mutations in ccRCC paired tissues with genetic test reports (*n* = 106). **I** Relationships between circPDHK1 expression and Ki-67 in ccRCC paired tissues (*n* = 112). **J** Illustration of circPDHK1 formation by splicing exons 2 and 8 from the PDHK1 parental gene as identified in CircBase (http://circrna.org). Specific divergent and convergent PCR primers to detect circPDHK1 and linear PDHK1 are indicated, respectively. **K** PCR products of circPDHK1 in cDNA and gDNA amplified using convergent or divergent primers in Caki-1 and 786-O. **L** Schematic illustration of circPDHK1 conformation. The exon 2–8 of PDHK1 mRNA formed PDHK1 through back splicing. As shown, Sanger sequencing was used to verify the back-splice junction site of circPDHK1. **M** Reverse transcription using oligo dT and random priming to identify circPDHK1 loop characteristics by RT-qPCR. **N** CircPDHK1 and linearPDHK1 mRNA stability was assessed using RNase R treatment. **O** RT-qPCR analysis of the abundance of circPDHK1 and PDHK1 linear mRNA in ccRCC cells treated with actinomycin D at the indicated time points. **P** RT-qPCR analysis of circPDHK1 location in the nucleus or cytoplasm in Caki-1 and 786-O cells. GADPH served as a marker of cytoplasmic location, while U6 served as a marker of nuclear location. **Q** Fluorescence in situ hybridization (FISH) was utilized to detect circPDHK1 localization in Caki-1 and 786-O cell lines. Bars = 20 μm. *P < 0.05; **P < 0.01; ***P < 0.001; ns, no significance

Our previous investigation successfully identified differentially expressed (DE) circRNAs in 4 pairs of ccRCC and adjacent noncancerous tissues (Supplementary Figure S1A) [11]. In the present study, we performed a WGCNA test to investigate the 3749 critical DE circRNAs and their host coding genes (Fig. 1A, B). Among the identified modules, the blue module consisted of 233 circRNAs that showed higher expression in ccRCC tissues than in noncancerous tissues (Supplementary Figure S1B-C and Table S6). To further screen candidate circRNAs, we intersected these 233 circRNAs with another ccRCC high-throughput circRNA microarray dataset (GSE100186) in the Gene Expression Omnibus (GEO) repository (Supplementary Figure S1D). We selected nine statistically significant and recurrently dysregulated circRNAs identified in both microarrays as validation candidates (Fig. 1C; Supplementary Table S7). Among these candidates, circPDHK1 (hsa_circ_0057090) showed the highest level of upregulation. This circRNA was formed by the back-splicing of exons 2–8 of the pyruvate dehydrogenase kinase 1 (PDHK1) gene.

Next, we evaluated the expression of circPDHK1 in ccRCC and paired noncancerous tissues from a cohort of 148 patients using RT-qPCR. We found that circPDHK1 expression was significantly increased in the cancerous tissues (Fig. 1D). Moreover, elevated circPDHK1 expression was observed in 84% (125/148) of the ccRCC patients (Supplementary Figure S1E). We further analyzed the relationship between circPDHK1 expression and clinicopathological features within the patient dataset. The ccRCC sample cohort sets (*n* = 148) were categorized based on WHO/ISUP stage, tumor T stage, and distant metastasis. Subsequently, we compared circPDHK1 expression levels between these groups (Supplementary Table S1). Briefly, higher circPDHK1 levels were associated with a higher WHO/ISUP grade (Fig. 1E), more advanced tumor T stage (Fig. 1F), and distant metastasis (Fig. 1G). Additionally, a statistical analysis of the genetic test reports of ccRCC patients (106/148) also indicated that the majority of patients possessed VHL mutations, and circPDHK1 levels were significantly higher in the VHL mutant group than in the VHL normal group (Fig. 1H). Furthermore, we correlated circPDHK1 expression levels with the clinical immunohistochemical index in 112/148 ccRCC patients. Significantly higher levels of circPDHK1 were confirmed in the Ki-67 ≥ 10% group than in the Ki-67 < 10% group (Fig. 1I). Moreover, circPDHK1 was significantly upregulated in ccRCC cell lines (Caki-1, 786-O, RCC-JF, and RCC-23) compared to the normal human kidney cell line HK-2. Among these ccRCC cell lines, Caki-1 cells exhibited the highest circPDHK1 expression, followed by 786-O cells (Supplementary Figure S1F). Collectively, these findings highlight the upregulation of circPDHK1 in ccRCC and suggest its potential role as a novel oncogene.

### Characterization of circPDHK1 in ccRCC

CircBase annotations also indicated that circPDHK1 (hsa_circ_0057090) was derived from exons 2 to 8 of the PDHK1 gene located on chromosome 2 q31.1 (Fig. 1J). To amplify circPDHK1 and linear PDHK1 from cDNA and gDNA, we constructed divergent and convergent primers. Our findings indicated that circPDHK1 was specifically amplified using cDNA, whereas linear PDHK1 mRNA was amplified from both cDNA and gDNA templates using convergent primers (Fig. 1K). Sanger sequencing confirmed the predicted splicing junction of circPDHK1 (Fig. 1L). Additionally, we conducted reverse transcription using random primers and observed the successful amplification of circPDHK1, further supporting its circular structure (Fig. 1M). Notably, circPDHK1 exhibited enhanced stability compared to linear PDHK1 after RNase R or actinomycin D treatment (Fig. 1N-O). In addition, subcellular fractionation and FISH assays demonstrated that circPDHK1 was predominantly cytoplasmic (Fig. 1P-Q). Overall, these findings demonstrated that circPDHK1 is a stable circRNA in ccRCC cells.

### Inhibition of circPDHK1 suppresses the proliferation, migration and invasion of ccRCC cells in vitro and in vivo

**Fig. 2:**
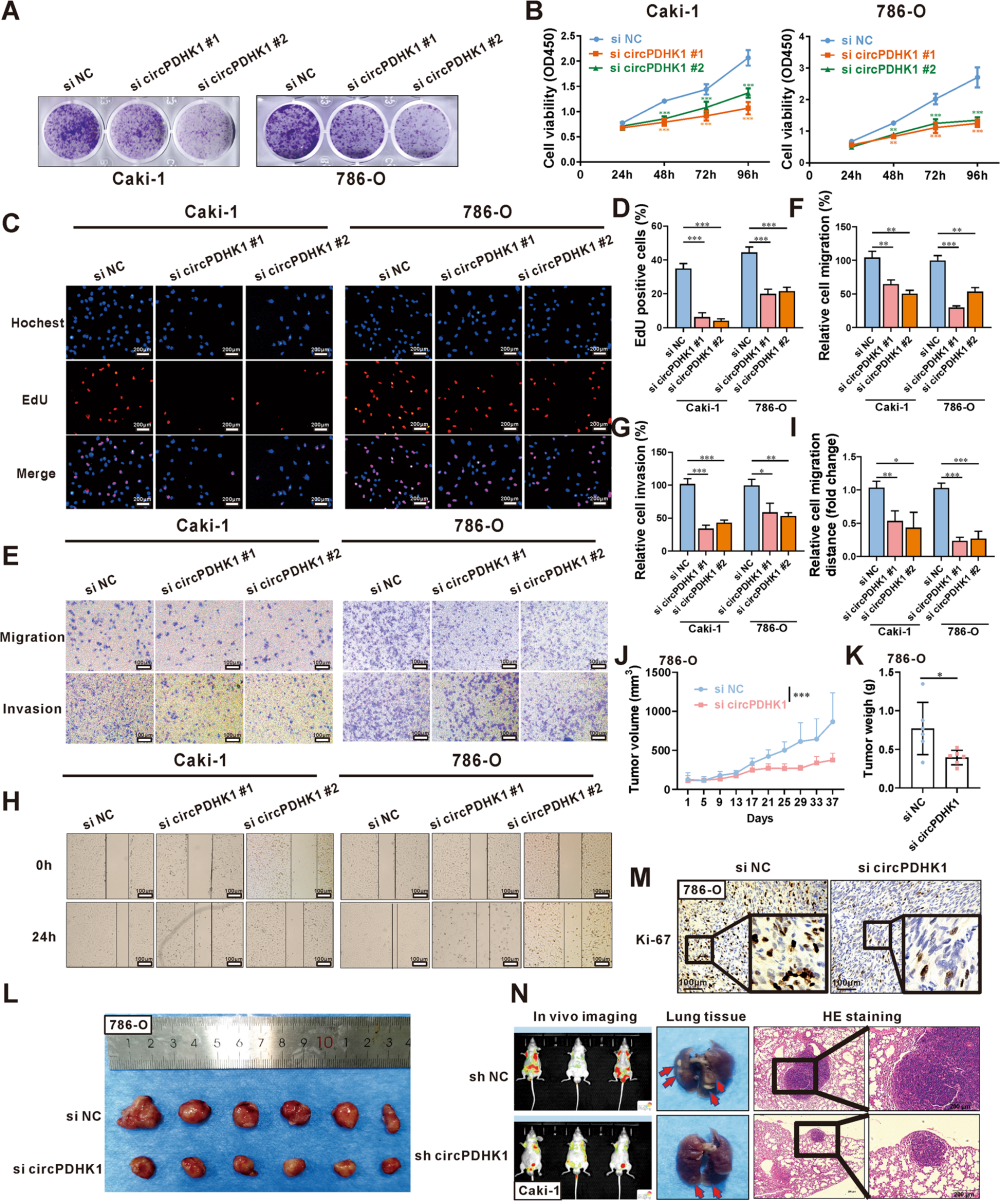
Inhibition of circPDHK1 suppresses the proliferation, migration and invasion of ccRCC cells in vitro and in vivo. **A** Colony formation assays, (**B**) CCK8 assays and (**C**, **D**) EdU assays were performed to detect the cell viability and proliferation activity of Caki-1 and 786-O cells transfected with two circPDHK1 siRNA. Bars = 200 μm. **E–G** Representative images and quantification of the transwell migration and invasion assays in circPDHK1 knockdown, and control ccRCC cells. Bars = 100 μm. **H-I** Representative images and quantification of the transwell migration and invasion assays in circPDHK1 knockdown, and control ccRCC cells. Bars = 100 μm. **J** Subcutaneous 786-O tumors in NSG mice injected with cholesterol-modified circPDHK1 siRNA, and tumor volumes were calculated every 2 d. At Day 37 after treatment, all mice were sacrificed, and (**K**) subcutaneous tumors were dissected and recorded. **L** Subcutaneous tumor was also photographed. (*n* = 6, each group). **M** Representative photomicrographs of Ki-67 immunohistochemical (IHC) staining of subcutaneous tumors. **N** We also established a lung metastasis model by injecting 6 × 10^6^ Caki-1 cells into the tail vein to evaluate the ability of cells to metastasize in vivo. Representative in vivo imaging of mice, photographs of whole lung tissues and hematoxylin–eosin (H&E) staining of lung metastatic nodules. (*n* = 3, each group) Bars = 200 μm. *P < 0.05; **P < 0.01; ***P < 0.001; ns, no significance

To explore the biological function of circPDHK1 in ccRCC cells, we constructed two siRNAs that targeted the splice junction site. These siRNAs were transfected into Caki-1 and 786-O cells, resulting in effective interference of circPDHK1 without altering linearPDHK1 mRNA expression (Supplementary Figure S2A-B). Subsequently, colony formation and CCK-8 and EdU assays were performed to assess the cell viability and proliferative capacity of Caki-1 and 786-O cells with circPDHK1 downregulation (Fig. 2A-D). Moreover, Transwell and wound-healing assays indicated that downregulation of circPDHK1 significantly inhibited migration and invasion abilities (Fig. 2E-I). Collectively, these results indicated that circPDHK1 accelerates the proliferation, migration, and invasion of ccRCC cells.

We verified the biological role of circPDHK1 in tumorigenicity using an in vivo model. NSG mice were used to establish an implanted tumor model using 786-O cells (Fig. 2J) and Caki-1 cells (Supplementary Figure S2C). Cholesterol-modified circPDHK1-siRNA was intratumorally injected, and the tumor status was monitored once every 2 days for 5 consecutive weeks for 786-O tumors and 3 consecutive weeks for Caki-1 tumors. Downregulation of circPDHK1 inhibited tumor growth and reduced tumor volume and weight (Fig. 2J-L; Supplementary Figure S2C-E). Furthermore, immunohistochemical analysis revealed decreased Ki-67 expression in tumors following circPDHK1 inhibition (Fig. 2M; Supplementary Figure S2F-G). Additionally, we investigated the metastatic potential of circPDHK1 in vivo by establishing a pulmonary metastasis model of ccRCC using Caki-1 cells stably transfected with circPDHK1 lentivirus (Supplementary Figure S2I). Six weeks after injection, the mice were assessed using an in vivo imaging system, and lung tissues were collected after the mice were sacrificed. The results demonstrated that mice injected with sh circPDHK1 Caki-1 cells exhibited reduced numbers and volumes of metastatic nodules compared to those in the control group (Fig. 2N).

Overall, our findings indicate that circPDHK1 promotes the proliferation, migration, and invasion of ccRCC cells both in vitro and in vivo.

### CircPDHK1 encodes a novel peptide, PDHK1-241aa

**Fig. 3:**
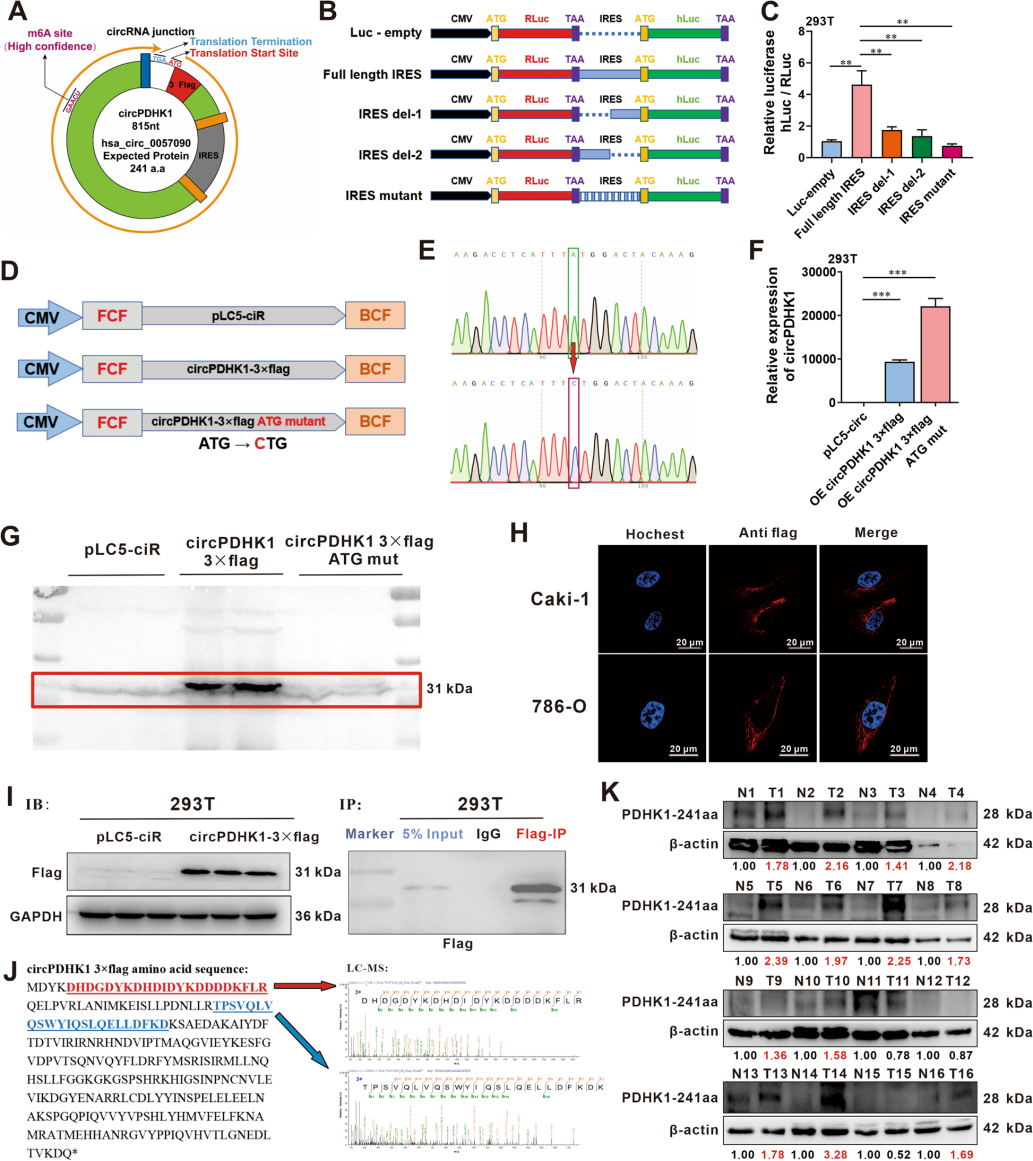
CircPDHK1 encodes a novel peptide, PDHK1-241aa. **A** The predicted 815 nt circPDHK1 sequence encodes 241 amino acids (PDHK1-241aa). Potential m6A sites and IRES (Internal Ribosome Entry Site) are also indicated. **B** IRES constructs in luciferase expression vectors. Full-length IRES, IRES del-1, IRES del-2 and mutated IRES types inserted in Rluc and hLuc reporter vectors containing independent start and stop codons. **C** Relative luciferase activity detected using dual-luciferase reporter assays to tested circPDHK1 translation ability in 293 T cells. **D** Illustration of the synthetic circRNA expression plasmid pLC5-ciR, a commercial circRNA blank expression vector; We also established circPDHK1-3 × flag plasmid for pLC5-circPDHK1 fused with 3 × flag tag and circPDHK1-3 × flag ATG mut, the start codon ATG of circPDHK1-3 × flag was mutated to CTG with a commercial kit to validate the coding potential of circPDHK1. **E** Sanger sequencing of the circPDHK1-3 × flag ATG mut vector to verify its construction. **F-G** RT‒qPCR and western blot were used to analyze the efficiency of overexpressing circPDHK1-3 × flag and circPDHK1-3 × flag ATG mut vectors to verified circPDHK1 translation ability in 293 T cells. **H** Immunofluorescence staining using a Flag antibody to detect the intracellular localization of circPDHK1-3 × flag in Caki-1 and 786-O cells. The red (anti-flag) indicated the circPDHK1-3 × flag, the blue (Hochest) indicated the nucleus. Bars = 20 μm. **I** PDHK1-241aa expression detected by western blot following SDS‒PAGE of IP pull-downs from cell lysates as indicated. Gel bands corresponding to 31 kDa were manually removed and submitted to LC‒MS for identification. **J** Identification of the amino acid sequence of PDHK1-241 by LC‒MS. **K** Western blot analysis of PDHK1-241aa expression in 16 paired ccRCC samples and normal adjacent tissues. **P < 0.01; ***P < 0.001

Prediction results from the circRNADb and Transcirc databases revealed that the circPDHK1 sequence contained an open reading frame (ORF) with an ATG initiation codon, an IRES at 112–261 nt, and several potential m6A modification sites. This indicated that circPDHK1 possessed the potential to encode a 241 aa peptide (Fig. 3A; Supplementary Figure S3A-C). To validate the activity of the predicted IRES in circPDHK1, we inserted it into a dual-luciferase vector and performed a luciferase reporter assay. The luciferase activity of the circPDHK1 full-length IRES reporter was significantly higher than that of the partial or mutated IRES reporters (Fig. 3B, C). Additionally, no significant enrichment of m6A modifications in circPDHK1 was observed in the meRIP assay. (Supplementary Figure S3D).

To further confirm the presence of PDHK1-241aa, we constructed circPDHK1 3 × flag-labeled vectors and mutated the predicted start codon ATG to CTG (ATG mut) in the circPDHK1 3 × flag overexpression plasmid (Fig. 3D, E). RT‒qPCR results revealed that cotransfection of circPDHK1 3 × flag and circPDHK1 3 × flag ATG mutant successfully overexpressed circPDHK1 in 293 T and ccRCC cells (Fig. 3F; Supplementary Figure S3E). However, flag-labeled PDHK1-241aa was only induced following circPDHK1 3 × flag transfection and not following transfection with the circPDHK1 3 × flag ATG mutant (Fig. 3G). Immunofluorescence staining using an anti-flag antibody confirmed the cytoplasmic localization of PDHK1-241aa 3 × flag in Caki-1 and 786-O cells overexpressing the circPDHK1 3 × flag plasmid (Fig. 3H). Furthermore, IP-MS assays using an anti-flag antibody in 293 T cells transfected with the circPDHK1 3 × flag vector confirmed the translation of PDHK1-241aa from circPDHK1. The PDHK1-241aa 3 × flag peptide migrated at the expected molecular weight of 31 kDa (Fig. 3I-J; Supplementary Figure S3F). Furthermore, we identified an antibody (NBP1-85955, Novus) that recognized the 92–151 aa region of PDHK1, coincidentally overlapping with the PDHK1-241 aa peptide (Supplementary Figure S3G). PDHK1–241 aa was highly expressed in 26 of the 32 paired ccRCC tissues (Fig. 3K; Supplementary Figure S3H) and cell lines (Supplementary Figure S3I) compared to the controls.

These analyses indicated that the annotated circPDHK1 encoded a novel peptide, PDHK1-241aa.

### PDHK1-241aa, not circPDHK1, promotes the proliferation, migration and invasion of ccRCC cells in vitro and in vivo

**Fig. 4:**
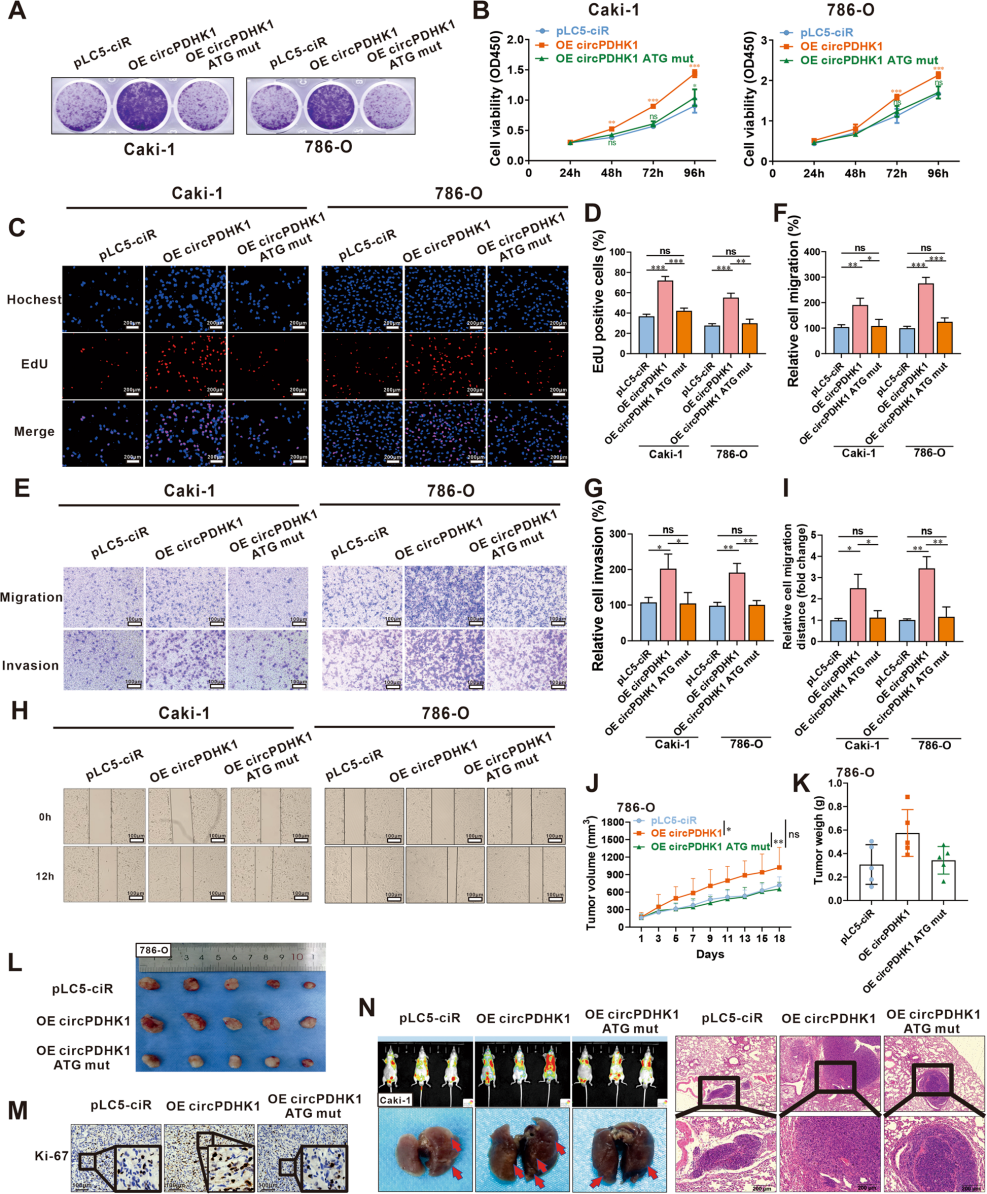
PDHK1-241aa, but not circPDHK1, promotes the proliferation, migration and invasion of ccRCC cells in vitro and in vivo. **A** Colony formation assays, **B** CCK-8 assays and (**C**, **D**) EdU assays were performed to detect the cell viability and proliferation activity of Caki-1 and 786-O cells transfected with pLC5-ciR, circPDHK1 and circPDHK1 ATG mut vectors. Bars = 200 μm. **E–G** Transwell assay to detect cell migration and invasion of Caki-1 and 786-O cells transfected with pLC5-ciR, circPDHK1 and circPDHK1 ATG mut vectors. Bars = 100 μm. **H**, **I** wound healing assays were conducted to evaluate cell migratory abilities in Caki-1 and 786-O cells tansfected with pLC5-ciR, circPDHK1 and circPDHK1 ATG mut vectors. Bars = 100 μm. **J** Xenograft 786-O tumors in NSG mice were injected with circPDHK1 and circPDHK1 ATG mut overexpression lentiviruses. Tumor volumes were calculated every 2 d. At Day 18 after treatment, all mice were sacrificed, and (**K**) subcutaneous tumors were dissected and recorded. **L** Subcutaneous tumor was also photographed. (*n* = 5, each group). **M** Representative photographs of Ki-67 IHC staining in subcutaneous tumors. **N** We also established a lung metastasis model by injecting 6 × 10^6^ Caki-1 cells into the tail vein to evaluate the ability of cells to metastasize in vivo. Representative in vivo imaging of mice, photographs of whole lung tissues and hematoxylin–eosin (H&E) staining of lung metastatic nodules. (*n* = 3, each group) Bars = 200 μm. *P < 0.05; **P < 0.01; ***P < 0.001; ns, no significance

To further investigate the biological function of PDHK1–241aa, we constructed vectors without the flag-tag that included circPDHK1, circPDHK1 ATG mut, and circPDHK1 CDS (Supplementary Figure S4A). The efficiency of circPDHK1 and its effect on PDHK1-241aa were confirmed by RT‒qPCR and western blotting (Supplementary Figure S4B-E). Colony formation, CCK8, and EdU assays demonstrated that transfection of circPDHK1 and the circPDHK1 CDS vector significantly enhanced cell viability and proliferation in ccRCC cells, in contrast to transfection of circPDHK1 ATG mut and the pCDH empty vector (Fig. 4A-D; Supplementary Figure S5A-D). In Transwell and wound healing assays, overexpression of circPDHK1 or circPDHK1 CDS accelerated the migration and invasion abilities of ccRCC cells, whereas overexpression of the circPDHK1 ATG mutant failed in these activities (Fig. 4E-I; Supplementary Figure S5E-I).

To further explore the potential tumor-promoting effect of PDHK1-241aa in vivo, we used NSG mice to establish an implanted tumor model using 786-O cells (Fig. 4J) and Caki-1 cells (Supplementary Figure S6). Lentiviral constructs containing circPDHK1 or the circPDHK1 ATG mutant were intratumorally injected, and tumor progression was monitored for 3 consecutive weeks. As expected, overexpression of circPDHK1 increased the volume and weight of xenograft tumors, whereas circPDHK1 ATG failed to accelerate tumor growth. (Fig. 4J-L; Supplementary Figure S6A-D). Additionally, IHC staining for the proliferation indicator Ki-67 revealed a significant increase in staining following circPDHK1 but not circPDHK1 ATG mut overexpression (Fig. 4M; Supplementary Figure S6E-F). For the pulmonary metastasis model, nude mice were injected with Caki-1 cells containing these constructs. The circPDHK1 group exhibited a higher number and larger volume of lung metastatic nodules than the circPDHK1 ATG mut group (Fig. 4N).

Collectively, these results demonstrated that the novel peptide PDHK1-241aa, but not circPDHK1, promoted the proliferation, migration, and invasion of ccRCC cells both in vitro and in vivo.

### PDHK1-241aa activates AKT-mTOR signaling in ccRCC cells

**Fig. 5:**
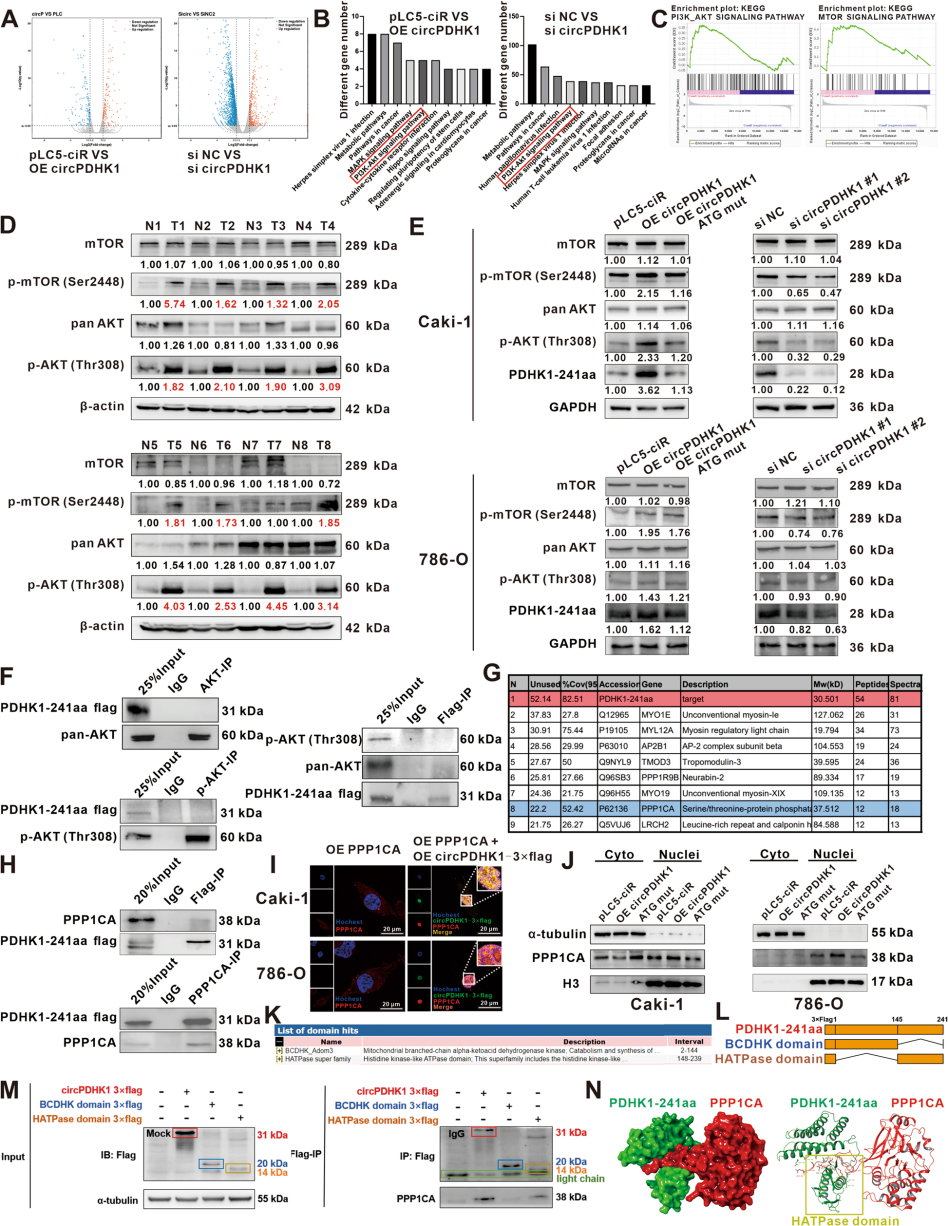
PDHK1-241aa interacts with PPP1CA to inhibit AKT dephosphorylation and activate the AKT-mTOR signaling pathway. **A** Volcano plot of differentially expressed protein-coding genes following circPDHK1 interference and overexpression. **B** The RNA-seq analysis showed that relevant KEGG enrichment pathways following circPDHK1 interference and overexpression in Caki-1 cells. **C** GSEA results plotted to visualize correlations between circPDHK1 and AKT-mTOR pathway expression in Caki-1 cells. Western blot detection of proteins related to the AKT-mTOR pathway in (**D**) 8 pairs of ccRCC tissues and (**E**) Caki-1 and 786-O cells following circPDHK1 interference and overexpression as indicated. **F** Coimmunoprecipitation (co-IP) assays were used to verify the interaction between PDHK1-241aa and AKT or p-AKT (Thr308). **G** Total proteins of PDHK1-241aa-flag-transfected HEK293T cells separated by SDS‒PAGE. 9 interacting proteins of circPDHK1 were identified by LC‒MS and listed with their corresponding scores. The LC‒MS analysis showed that identification of PPP1CA in the PDHK1-241aa protein complex. **H** Co-IP used to verify binding between PPP1CA and PDHK1-241aa. **I** Immunofluorescence localization of PDHK1-241aa-flag and PPP1CA from overexpression vectors in Caki-1 and 786-O cells. The green (anti-flag) indicated the circPDHK1-3 × flag; The red(anti-PPP1CA) indicated the PPP1CA; The blue (hochest) indicated the nucleus. Bars = 20 μm. **J** Variations in PPP1CA and PDHK1-241aa localization verified by nuclear and cytoplasmic separation assays. **K** NCBI database (https://www.ncbi.nlm.nih.gov/cdd/) predicted that the PDHK1 domain is composed of BCDHK domain and HATPase domain, respectively. **L** Schematic illustrations of PDHK1 expression plasmids. **M** We also established truncation mutants BCDHK domain 3 × flag and HATPase domain 3 × flag plasmid. Immunoblot analysis following cotransfection with PDHK1-241aa and the indicated truncation mutants. **N** The specific binding between PDHK1-241aa and PPP1CA was predicted by PyMOL (https://pymol.org/2/) and HDOCK (http://hdock.phys.hust.edu.cn/). *P < 0.05; **P < 0.01; ***P < 0.001; ns, no significance

We further explored the potential regulatory mechanisms of PDHK1-241aa in ccRCC cells. RNA-seq analyses were performed on Caki-1 cells transfected with pLC5-circPDHK1 and si circPDHK1 (Fig. 5A). Differential expression analysis was conducted, and the top 10 significantly differentially expressed genes (DEGs) were identified, along with their associated signaling pathways (Fig. 5B). Gene set enrichment analysis (GSEA) further demonstrated a correlation between circPDHK1 expression and the AKT-mTOR signaling pathway (Fig. 5C). This pathway predominantly functions in tumor growth and metastasis [33, 34]. Therefore, we further assessed AKT-mTOR activation in eight paired ccRCC tissues. We observed a significant increase in the phosphorylation levels of p-AKT (Thr308) and p-mTOR (Ser2448) compared to those in adjacent noncancerous tissues (Fig. 5D). We also examined whether circPDHK1 or circPDHK1 ATG mutant overexpression or inhibition (si circPDHK1) altered AKT-mTOR phosphorylation. Overexpression of circPDHK1 in Caki-1 and 786-O cells led to increased phosphorylation of p-AKT (Thr308) and p-mTOR (Ser2448), which was correlated with upregulated PDHK1-241aa expression. In contrast, overexpression of circPDHK1 ATG failed to alter the phosphorylation levels. Moreover, siRNA-mediated inhibition of circPDHK1 resulted in the downregulation of PDHK1-241aa and decreased phosphorylation levels of p-AKT (Thr308) and p-mTOR (Ser2448) (Fig. 5E). In addition, it is noteworthy that there was no significant change in other proteins related to AKT-mTOR pathway such as PI3K, p-PI3K, PDK1, PTEN in Caki-1 and 786-O cells after silencing or overexpression of circPDHK1 (Supplementary Figure S7). Collectively, these results illustrate that PDHK1-241aa but not circPDHK1 acts as a regulator of AKT-mTOR phosphorylation in ccRCC, and PDHK1-241aa does not activate AKT by affecting its upstream proteins.

In addition, we used LY-294002 (AKT inhibitor) and Rapamycin (mTOR inhibitor) to evaluate the effects of inhibitors alone or in combination with overexpression of PDHK1-241aa on the tumor biological behavior of ccRCC cells. We demonstrated that LY-294002 (20 μM), and Rapamycin (10 μM) suppressed the capacity of proliferation, migration and invasion in Caki-1 and 786-O cells by using CCK8, EdU, and transwell assays (Supplementary Figure S8-9). Most notably, treatment with above inhibitors significantly counteracted circPDHK1-overexpression-induced enhancement of proliferation and migration in ccRCC cells. Above data suggest that the function of PDHK1-241aa depends on AKT-mTOR signaling pathway to some extent.

### PDHK1-241aa interacts with PPP1CA to inhibit AKT dephosphorylation and activate the AKT-mTOR signaling pathway

AKT phosphorylation is a critical initial step in AKT-related signaling pathways. We verified that PDHK1-241aa increased the phosphorylation of AKT Thr308. However, Co-IP experiments did not confirm the interaction between PDHK1-241aa and AKT or p-AKT (Thr308) (Fig. 5F). To explore the underlying mechanism of this novel peptide, we screened for potential binding proteins of PDHK1-241aa using LC‒MS (Supplementary Table S8). We identified PPP1CA in the group of most abundant proteins, which functions as a serine/threonine-protein phosphatase and possesses a high binding coefficient with PDHK1-241aa (Fig. 5G). Importantly, AKT is a known target of PPP1CA [26, 28]. Co-IP experiments confirmed the interaction between PDHK1-241aa and PPP1CA (Fig. 5H). RT‒qPCR and western blotting results demonstrated that circPDHK1 or PPP1CA had no effect on RNA or protein levels of each other (Supplementary Figure S10A-B). Then we evaluated the expression of PPP1CA in ccRCC tissues. Consistent with our validation results (*n* = 24), TCGA database also showed that there was no significant difference in PPP1CA expression between ccRCC tissues and noncancerous tissues. (Supplementary Figure S10C-E). Interestingly, immunofluorescence experiments revealed that PPP1CA was localized in both the cytoplasm and nucleus and that PDHK1-241aa was primarily cytoplasmic. However, PPP1CA and PDHK1-241aa colocalized in the nucleus after the co-overexpression of PPP1CA and PDHK1-241aa, suggesting that the binding between PPP1CA and PDHK1-241aa may result in their nuclear translocation (Figs. 3H and 5I; Supplementary Figure S10F). Moreover, extraction of nucleoplasmic proteins further confirmed that PDHK1-241aa overexpression resulted in increased PPP1CA levels in the nucleus and decreased PPP1CA levels in the cytoplasm (Fig. 5J). Therefore, we hypothesized that PDHK1-241aa could bind to PPP1CA and alter the localization of PPP1CA and PDHK1-241aa. This results in decreased AKT dephosphorylation, leading to an increase in AKT phosphorylation in ccRCC.

The predicted PDHK1-241aa structure possessed two functional domains: BCDHK and HATPase (Fig. 5K). Consequently, we generated two segmented truncated vectors containing individual BCDHK or HATPase domains to assess their functional activities (Fig. 5L). Co-IP assays using anti-flag and anti-PPP1CA antibodies demonstrated that the HATPase domain of PDHK1-241aa was responsible for the interaction between PDHK1-241aa and PPP1CA (Fig. 5M; Supplementary Figure S10G). Additionally, we used PyMOL and HDOCK software to predict the docking structure between PDHK1-241aa and PPP1CA and found that the HATPase domain was the main structure binding to PPP1CA (Fig. 5N).

**Fig. 6:**
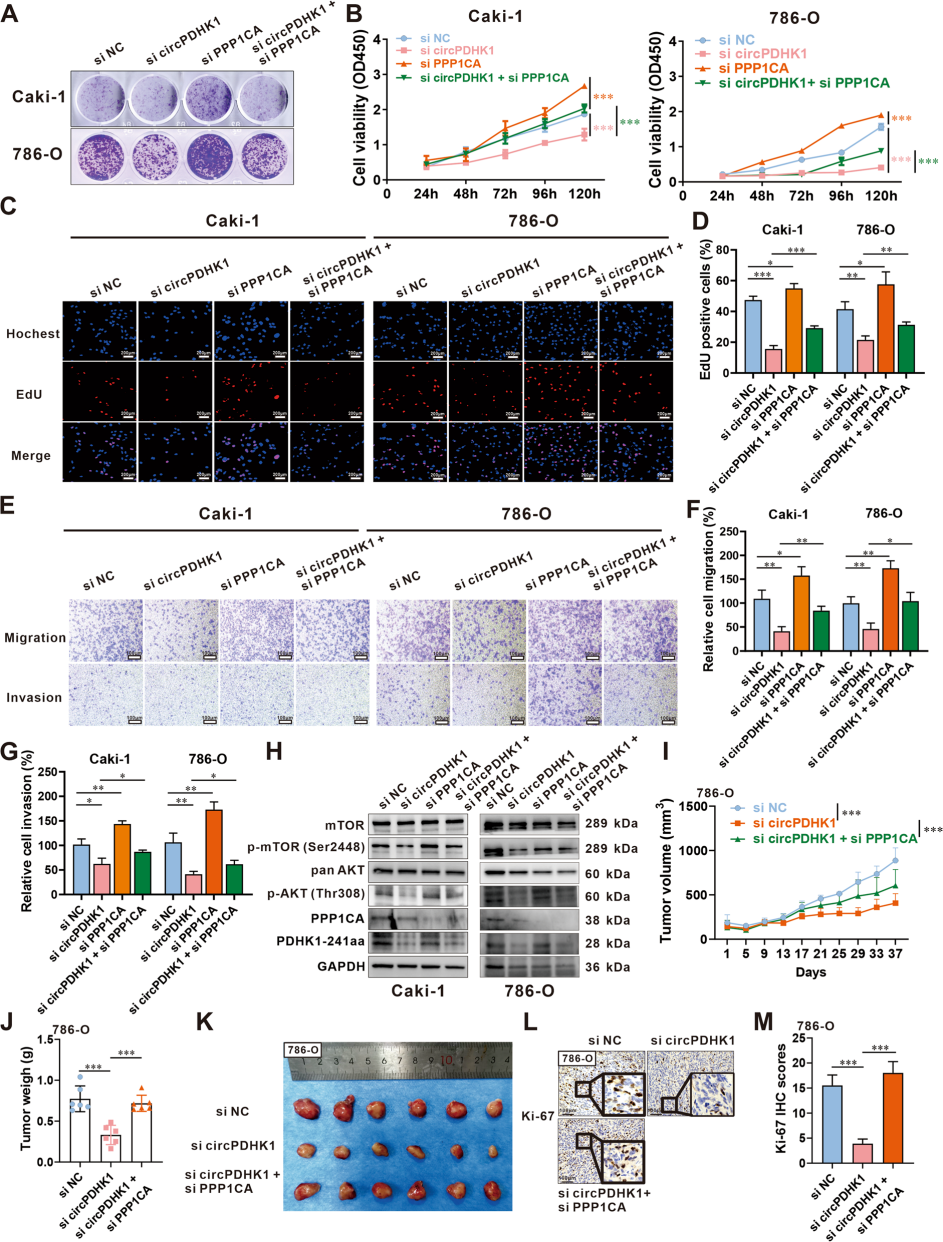
Phenotypes of Caki-1 and 786-O cells cotransfected with siRNAs for circPDHK1 and PPP1CA. **A** Colony formation assays, (**B**) CCK-8 assays and (**C**, **D**) EdU assays were performed to detect the cell viability and proliferation activity of Caki-1 and 786-O cells transfected with circPDHK1 siRNA and/or PPP1CA siRNA. Bars = 200 μm. **E–G** Transwell migration and invasion assays to detect cell migration ability of Caki-1 and 786-O cells transfected with the control siRNA, a circPDHK1 siRNA, a PPP1CA knockdown siRNA and cotransfected with the circPDHK1 siRNA and the PPP1CA knockdown siRNA. Bars = 100 μm. **H** Western blot detection of the phosphorylation levels of the AKT-mTOR signaling pathway in Caki-1 and 786 cells. **I** Analysis of subcutaneous tumors in mice injected with cholesterol-modified the control siRNA, a circPDHK1 siRNA and cotransfected with the circPDHK1 siRNA and the PPP1CA knockdown siRNA. Tumor volumes calculated every 2 d. **J** At Day 37 after treatment, all mice were sacrificed, and subcutaneous tumors were dissected and recorded. Tumor weights in each group as indicated. **K** Images of subcutaneous tumors. (*n* = 6, each group). **L**, **M** Representative photographs and quantification of Ki-67 IHC staining in subcutaneous tumors. *P < 0.05; **P < 0.01; ***P < 0.001; ns, no significance

Subsequently, we performed rescue experiments to investigate the effect of the interactions between PDHK1-241aa and PPP1CA on the malignant phenotypes of ccRCC cells. Successful knockdown (siRNA) and overexpression of PPP1CA were confirmed (Supplementary Figure S11A-B). Colony formation, CCK-8, EdU and Transwell assays revealed that downregulation of PDHK1-241aa significantly suppressed the proliferation, migration, and invasion of ccRCC cells, whereas downregulation of PPP1CA exhibited the opposite pattern. Rescue experiments demonstrated that the coinhibition of PDHK1-241aa and PPP1CA counteracted their respective effects on ccRCC cells (Fig. 6A-G). In contrast, overexpression of PDHK1-241aa significantly promoted the proliferation, migration, and invasion of ccRCC cells, whereas overexpression of PPP1CA had the opposite effect. Rescue experiments also indicated that the co-overexpression of PPP1CA and circPDHK1 significantly impaired the circPDHK1-induced enhancement of proliferation, migration, and invasion in ccRCC cells (Supplementary Figure S12A-G).

Additionally, we verified the phosphorylation levels of the AKT-mTOR signaling pathway in ccRCC cells with cointerference or cooverexpression of PDHK1-241aa and PPP1CA. The inhibition of circPDHK1 downregulated PDHK1-241aa expression and decreased p-AKT (Thr308) and p-mTOR (Ser2448) levels. Conversely, circPDHK1 overexpression increased the expression of PDHK1-241aa, p-AKT (Thr308), and p-mTOR (Ser2448). Moreover, siRNA targeting PPP1CA elevated the phosphorylation levels of p-AKT (Thr308) and p-mTOR (Ser2448), whereas PPP1CA overexpression showed the opposite effect. Rescue experiments showed that the phosphorylation levels of AKT-mTOR signaling pathway proteins could be restored by cointerference or cooverexpression of PDHK1-241aa and PPP1CA (Fig. 6H; Supplementary Figure S12H).

Next, we established an implanted tumor model using 786-O cells in mice and intratumorally injected cholesterol-modified circPDHK1-siRNA and PPP1CA-siRNA. We observed that silencing PPP1CA inhibited circPDHK1-siRNA-induced downregulation of tumor growth in subcutaneously implanted tumors, resulting in decreased tumor volumes and weights (Fig. 6I-L; Supplementary Figure S12I).

In conclusion, our findings suggest that PDHK1-241aa interacts with PPP1CA to attenuate AKT dephosphorylation, consequently activating the AKT-mTOR signaling pathway. PDHK1-241aa may facilitate ccRCC proliferation and metastasis.

### CircPDHK1 silencing improves the sensitivity of ccRCC to small-molecule targeted drugs

Given the high involvement of AKT-mTOR signaling in dictating cancer resistance to both small-molecule targeted drugs and biological agents [35], we evaluated the impact of silencing of circPDHK1 (si-circPDHK1) on the anti-tumor efficacy of tyrosine kinase inhibitors (TKI), PI3K-AKT inhibitor, or mTOR inhibitor. Firstly, we compared the effects of Pazopanib or Sunitinib alone and in combination with si-circPDHK1 on ccRCC cells viability. We found that the half-maximum inhibitory concentration (IC50) of Pazopanib or Sunitinib combined with si-circPDHK1 was lower than that in Pazopanib or Sunitinib alone group, proving that circPDHK1 silencing improved the sensitivity of ccRCC to TKI drugs to a certain extent (Supplementary Figure S13A). Secondly, we evaluated the effects of Pazopanib alone, LY-294002 (AKT inhibitor) alone, Rapamycin (mTOR inhibitor) alone or in combination with si-circPDHK1 on the tumor biological behavior of ccRCC cells through CCK8, EdU, and transwell assays. When Caki-1 and 786-O cells were treated with Pazopanib, LY-294002, or Rapamycin, the proliferation and migration ability were significantly inhibited, while in combination with si-circPDHK1 exacerbated the inhibition of cell proliferation and migration to some extent (Supplementary Figure S13B, S14 and S15).

Collectedly, our data suggest silencing circPDHK1 can partially enhance the anti-tumor efficacy and sensitivity of TKI drugs or AKT-mTOR inhibitors for ccRCC in vitro*.*

### Biogenesis of circPDHK1 is activated by HIF-2A

Given that the biogenesis of some circRNAs initiates from and is coupled with the transcription of circRNA-producing pre-mRNAs, we speculated that the generation of circPDHK1 might be associated with its parent linear PDHK1. Recently, another circRNA derived from PDHK1 (hsa_circ_0057104) and its pre-PDHK1 was identified to be transcriptionally activated by HIF-1A under hypoxic conditions [36]. However, no significant difference in HIF-1A levels was observed in ccRCC samples from the TCGA database (Supplementary Figure S16A). Moreover, previous studies have reported that HIF-1A functions as a tumor suppressor in ccRCC, whereas HIF-2A has been implicated as an oncoprotein in ccRCC [20]. HIF-2A can initiate the transcription of many disease-related genes at high levels in ccRCC [22, 37]. Therefore, we aimed to investigate whether the elevated levels of circPDHK1 in ccRCC were accompanied by elevated transcription of its host gene, PDHK1, which is activated by HIF-2A.

**Fig. 7:**
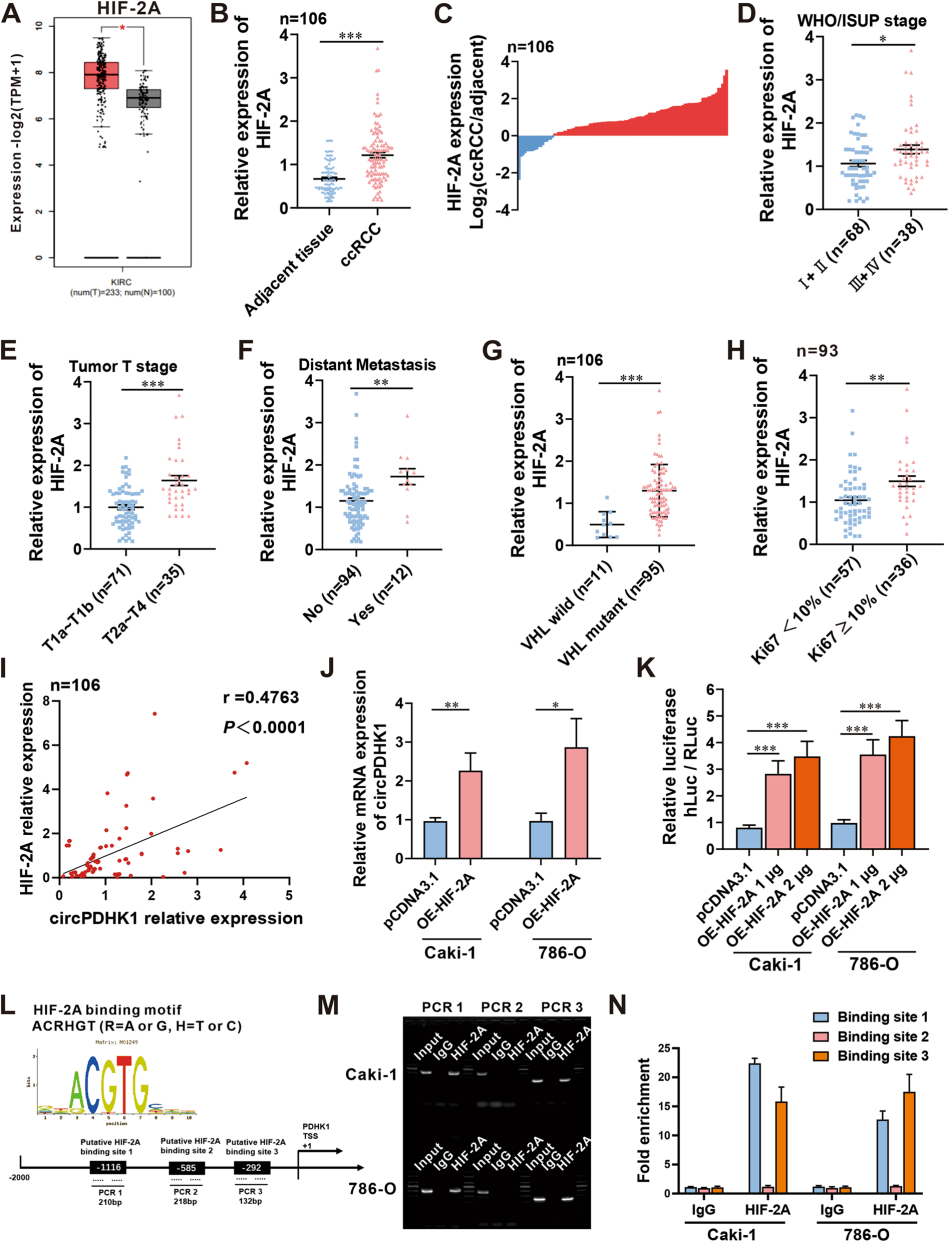
Biogenesis of circPDHK1 is activated by HIF-2A. **A** HIF-2A expression data in KIRC samples and normal adjacent tissues from TGCA. **B**, **C** RT-qPCR to detected relative HIF-2A expression in 106 paired ccRCC tissues and normal adjacent tissues. **D** Comparison of HIF-2A expression between patients with WHO/ISUP stage III–IV (*n* = 38) and those with WHO/ISUP stage I–II (*n* = 68), detected by RT-qPCR. **E** Comparison of HIF-2A expression between patients with tumor T stage T1a-T1b (*n* = 71) and those with tumor T stage T2a-T4 (*n* = 35), detected by RT-qPCR. **F** Association analyses of HIF-2A expression and distant tumor metastasis. **G**, **H** Relationships between HIF-2A expression and VHL mutation (*n* = 106) and Ki-67 expression (*n* = 93). **I** RT-qPCR validation of correlation between circPDHK1 and HIF-2A expression in ccRCC paired tissues (*n* = 106). **J**, **K** Regulatory relationships between circPDHK1 and HIF-2A using RT‒qPCR detection and dual-luciferase assays in Caki-1 and 786-O cells. **L** Schematic of the HIF-2A binding motif and the HREs in the PDHK1 promoter obtained from the Contra V3 database (http://bioit2.irc.ugent.be/contra/v3) as indicated. We also designed three primers for the different binding sites in PDHK1 promoter. **M**, **N** ChIP assay to assess HIF-2A binding site interactions in the PDHK1 promoter region. *P < 0.05; **P < 0.01; ***P < 0.001; ns, no significance

We first analyzed HIF-2A expression in 106 ccRCC patients and compared these data with genetic test reports. Consistent with the TCGA database, we observed a significant upregulation of HIF-2A expression in ccRCC tissues compared to that in adjacent noncancerous tissues (Fig. 7A-C). Subsequently, we investigated the association between HIF-2A expression and clinicopathological features by categorizing the ccRCC sample cohort based on the WHO/ISUP stage, tumor T stage, and distant metastasis and compared HIF-2A levels (Supplementary Table S9). Briefly, higher levels of HIF-2A were associated with a higher WHO/ISUP grade, more advanced tumor T stage, and distant metastasis in ccRCC (Fig. 7D-F). Additionally, we observed significantly elevated levels of HIF-2A in the VHL mutant group compared with those in the VHL wild-type group (Fig. 7G). Furthermore, we correlated HIF-2A expression levels with the clinical immunohistochemical index of Ki-67 expression. We found that in 93 samples with Ki-67 staining out of 106 ccRCC patients, HIF-2A levels were significantly higher in the Ki-67 ≥ 10% group than in the Ki-67 < 10% group (Fig. 7H). Pearson’s correlation analysis demonstrated a positive correlation between circPDHK1 and HIF-2A mRNA expression levels (Fig. 7I). These results indicated that HIF-2A is overexpressed in ccRCC cells. Thus, we hypothesized that upregulation of circPDHK1 may be related to HIF-2A activity. To validate this hypothesis, we transfected an HIF-2A overexpression plasmid into ccRCC cells and observed a significant increase in circPDHK1 expression (Fig. 7J). Instead, PT-2385 (HIF-2A inhibitor) suppressed the expression of circPDHK (Supplementary Figure S8B). Subsequently, a dual-luciferase reporter assay was performed in 293 T cells, and luciferase activity was enhanced by a reporter containing the PDHK1 promoter sequence when HIF-2A was overexpressed (Fig. 7K). Promoter sequence analysis tools (Contra Ver. 3) identified three potential HIF-2A hypoxia response elements (HREs) (ACRHGT, R = A or G, and H = T or C) in the genomic promoter regions of circPDHK1 (Fig. 7L). The direct interaction of HIF-2A with these binding sites was confirmed by ChIP-qPCR. We found that HIF-2A could directly bind to sites 1 and 3 of the genomic PDHK1 promoter region (Fig. 7M, N).

**Fig. 8:**
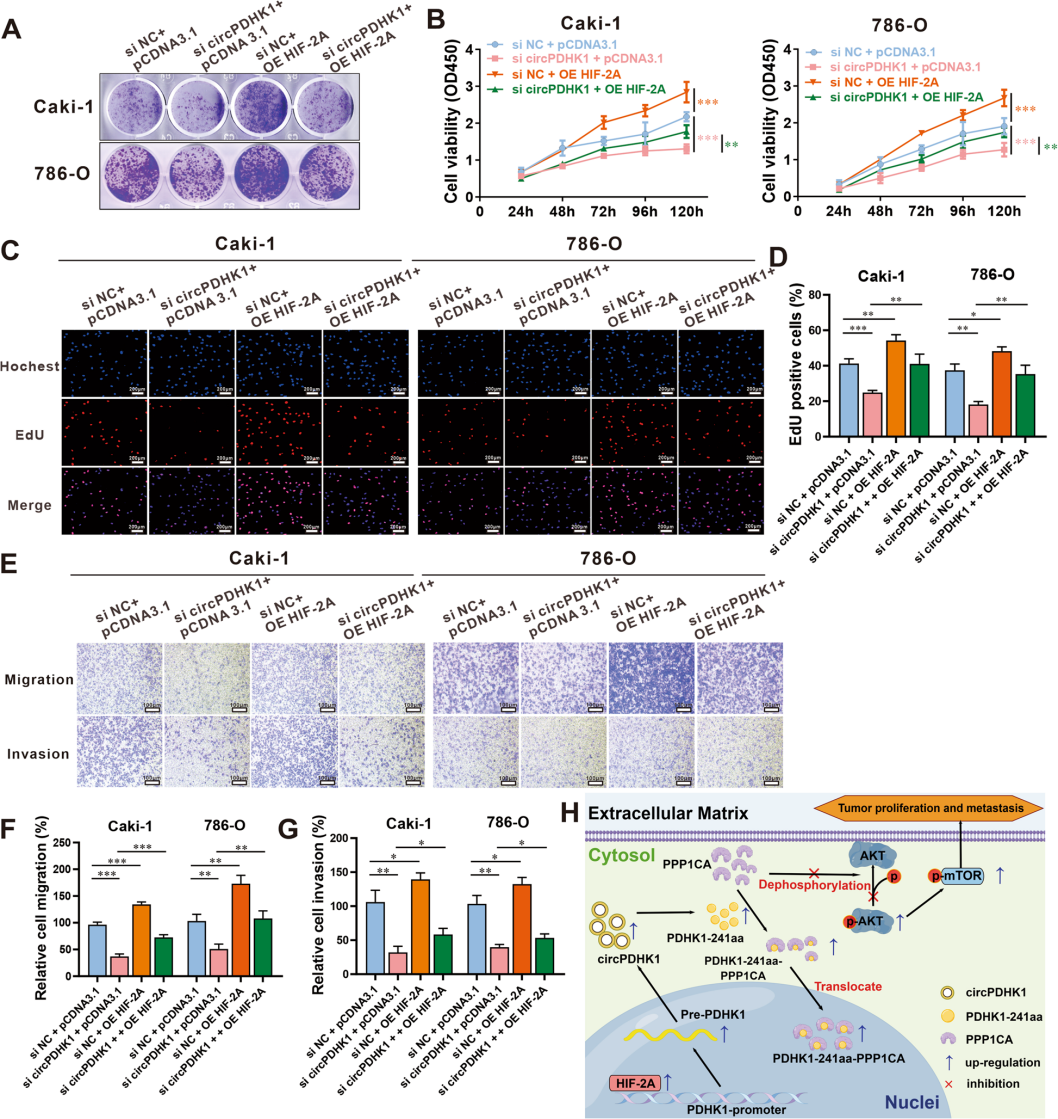
Cotransfection analysis of circPDHK1 siRNA and HIF-2A overexpression vector in Caki-1 and 786-O cells. **A** Colony formation assays, (**B**) CCK-8 assays and (**C**, **D**) EdU assays were performed to detect the cell viability and proliferation activity of Caki-1 and 786-O cells transfected with circPDHK1 siRNA and/or HIF-2A overexpression vector. Bars = 200 μm (**E–G**) Transwell migration and invasion assays to detect cell migration ability of Caki-1 and 786-O cells cotransfected with the control siRNA and pCDNA3.1 vector, a circPDHK1 siRNA and pCDNA3.1 vector, the control siRNA and HIF-2A overexpressed vector, the circPDHK1 siRNA and the HIF-2A overexpressed vector. Bars = 100 μm. **H** Graphical depiction of circPDHK1 encoding PDHK1-241aa, which binds to PPP1CA to inhibit AKT dephosphorylation and activate the AKT-mTOR signaling pathway, thereby promoting the proliferation and metastasis of ccRCC. The schematic was drawn by Figdraw (https://www.figdraw.com). *P < 0.05; **P < 0.01; ***P < 0.001; ns, no significance

Subsequently, we employed cell phenotypic assays, including colony formation, CCK-8, EdU, and Transwell assays, to investigate whether circPDHK1 function was HIF-2A dependent. We confirmed the successful regulation of circPDHK1 and HIF-2A using our experimental system (Supplementary Figure S16B). These findings demonstrated that downregulation of circPDHK1 suppressed the proliferation, migration, and invasion abilities of ccRCC cells, whereas overexpression of HIF-2A produced the opposite results. Rescue experiments also indicated that circPDHK1 inhibition partially restored the HIF-2A-induced proliferation, migration, and invasion abilities of ccRCC cells (Fig. 8A-G).

In addition, we evaluated the effects of PT-2385 alone or in combination with overexpression of circPDHK1 on the tumor biological behavior of ccRCC cells. We demonstrated that PT-2385 (10 μM) suppressed the capacity of proliferation, migration and invasion of Caki-1 and 786-O cells. Most notably, PT-2385 significantly counteracted circPDHK1-overexpression-induced enhancement of proliferation and migration in ccRCC cells (Supplementary Figure S8C and S9).

Collectively, these results indicate that the upregulation of circPDHK1 in ccRCC was due in part to HIF-2A activation.

## Discussion

Accumulating evidence suggests that circRNAs play crucial roles in biological regulatory networks and are closely associated with various diseases, including liver, breast, and esophageal cancers and other carcinomas [38–40]. However, only a few circRNAs have been implicated in ccRCC. For example, circME1 promotes aerobic glycolysis in ccRCC cells by enhancing the expression of its parental gene ME1, which results in ccRCC progression and the development of sunitinib resistance [41]. Additionally, circPPP6R3 serves as a miR-1238-3p sponge, resulting in the upregulation of CD44 expression, thereby modulating the proliferation, migration, and invasion of ccRCC [42]. Our previous study also found that circTNPO3 suppressed the proliferation and migration of ccRCC cells by directly interacting with IGF2BP2 and synergistically destabilizing SERPINH1 mRNA [11]. Exploration of circRNA-based targeted therapeutic strategies has provided new insights into the treatment of ccRCC. Therefore, it is necessary to identify additional functional circRNAs in ccRCC.

Herein, we identified, for the first time, that circPDHK1 derived from exons 2 to 8 of PDHK1 exhibited high expression levels in ccRCC. Importantly, its expression levels were positively correlated with the WHO/ISUP stage, T stage, and M stage in ccRCC patients. Our functional investigations revealed that circPDHK1 promotes the proliferation and metastasis of ccRCC cells. CircRNAs derived from the PDHK1 host gene are implicated in tumorigenesis and metastasis. For instance, hsa_circ_0057104, an alternative splicing isoform of PDHK1, modulates the miR-628-3p/BPTF axis and degrades BIN1, thereby enhancing the growth, metastasis, and glycolysis of pancreatic cancer cells [36]. CircPDHK1 (hsa_circ_0118104) is significantly correlated with tumor metastasis through the circPDHK1-miR-377-3P-NOTCH1 axis [43]. These findings collectively demonstrate that circPDHK1 plays a pivotal role in ccRCC progression and is a potential biomarker for the diagnosis and prognosis of ccRCC.

Multiple studies have highlighted the significant roles of circRNAs in various functional mechanisms, including the miRNA sponge effect, interaction with RNA-binding proteins, formation of R-loops, and translation of functional proteins [44, 45]. Although circRNAs have been defined as noncoding RNAs, some possess IRES or m^6^A modifications and are no longer noncoding RNAs in the traditional sense but are special circular RNAs capable of encoding peptides through multiple mechanisms [46–49]. We conducted a study based on the prediction results obtained using the circRNADb and Transcirc databases. Our findings revealed that circPDHK1 is translatable via an IRES and encodes a novel isoform, PDHK1-241aa. We confirmed that PDHK1-241aa was highly expressed in ccRCC tissues and predominantly present in the cytoplasm. We also showed that the PDHK1-241aa protein is the regulatory molecule involved in ccRCC cell proliferation and metastasis, since the nontranslatable ATG mut mutant was inactive. Thus, circPDHK1 can improve the proliferation and metastasis of ccRCC cells by encoding PDHK1-241aa, but further validation of the underlying mechanism is needed.

Emerging studies have identified the role of peptides encoded by circRNAs in tumorigenesis and cancer development. For instance, circHER2 encodes a novel protein, HER2-103aa, which forms a dimer with EGFR to phosphorylate AKT (Thr308) in certain triple-negative breast cancers (TNBCs). HER2-103aa promotes TNBC cell proliferation, invasion and tumorigenesis [50]. CircSEMA4B encodes SEMA4B-211aa, which is expressed at a low level in breast cancer and exerts a tumor suppressor function in vivo and in vitro by complexing with free p85 to decrease the p85/p110 PI3-kinase complex, leading to the inhibition of AKT (Thr308) phosphorylation and the generation of PIP3 [51]. In the present study, we observed a positive correlation between PDHK1-241aa levels and ccRCC cell proliferation and metastasis. Furthermore, these levels were associated with positive regulation of phosphorylation within the AKT-mTOR signaling pathway.

Multiple studies have shown that AKT-mTOR signaling is a promising target for cancer therapy [33, 52, 53]. This pathway regulates cancer cell survival and growth, and mTOR is a direct substrate of AKT kinase. Augmented activation or amplification of the AKT-mTOR pathway inhibits cell apoptosis, accelerates cell proliferation, and induces metastasis. The hyperactivation of AKT has been implicated in various pathophysiological conditions [54, 55]. Generally, AKT undergoes dual phosphorylation, and its PH domain binds to PIP3 and translocates to the cell membrane. Thr308 and Ser473 are two important phosphorylation sites for AKT [56, 57]. Given that kinases, such as 3-phosphoinositide-dependent protein kinase 1 (PDPK1), are also recruited to the membrane via their PH domain and phosphorylate AKT at Thr308 in its active loop [56, 58], we wondered whether PDHK1-241aa has a PH-like domain to phosphorylate AKT. Interestingly, our findings confirmed that PDHK1-241aa increases the phosphorylation of p-AKT (Thr308) and p-mTOR (Ser2448). However, the co-IP and LC‒MS data indicated that PDHK1-241aa did not bind to AKT or p-AKT (Thr308). Therefore, PDHK1-241aa indirectly regulates AKT phosphorylation.

We further explored the mechanism of PDHK1-241aa function and screened for potential interacting proteins using PDHK1-241aa-flag IP assays and LC‒MS. We focused on the serine/threonine-protein phosphatase PPP1CA (PP1-α catalytic subunit), which is closely associated with phosphorylation patterns and plays a significant role in regulating cellular processes [28, 59, 60]. For instance, PPP1CA physically interacts with the NACA protein, dephosphorylating NACA to enhance c-Jun activity in osteoblasts [60]. PPP1CA is a major phosphatase that can directly dephosphorylate AKT to regulate cell survival and differentiation [27]. Through co-IP experiments, we confirmed that PDHK1-241aa directly binds to PPP1CA and inhibits AKT dephosphorylation. Interestingly, the interaction between PDHK1-241aa and PPP1CA promoted nuclear aggregation of PPP1CA, suggesting that PDHK1-241aa translocates PPP1CA from the cytoplasm to the nucleus. PPP1CA has been shown to shuttle between the nucleus and cytosol [61]. Similarly, the small nucleolar RNA (snoRNA) SNORD12B can bind to PPP1CA and relocate it from the cytosol to the nucleus by disrupting the interactions between 14–3-3ζ and PPP1CA. This relocation of PPP1CA enhances AKT phosphorylation in the cytosol, activates the AKT-mTOR signaling pathway, and contributes to esophageal cancer tumorigenesis [28]. Thus, our findings provide a novel mechanism by which PDHK1-241aa inhibits the inactivation of the AKT-mTOR signaling pathway. Furthermore, multiple studies have shown that abnormal activation of the AKT-mTOR axis is a vital cause of tyrosine kinase inhibitors (TKI) resistance in ccRCC [62]. Due to the regulatory effect of circPDHK1 on AKT-mTOR signaling pathway, we discovered that silencing circPDHK1 can partially enhance the anti-tumor efficacy and sensitivity of TKI drugs or AKT-mTOR inhibitors for ccRCC in vitro.

CircRNA biogenesis regulation is complex [63–65]. In addition to back-splicing factors, transcription regulatory factors also affect the abundance of circRNAs [66]. It has been reported that circRNA expression can be modulated through activation of host gene transcription. For example, Twist1 promotes Cul2 transcription by binding to the Cul2 promoter. This process upregulates circCul2 expression to accelerate endothelial-mesenchymal transition (EMT) in hepatocellular carcinoma [67]. Sp1 directly binds to the B3 region of the Hipk2 promoter and upregulates circHipk2 and linear Hipk2 expression in myoblasts [68]. Interestingly, hsa_circ_0057104, which is also derived from PDHK1, was activated by HIF-1A at the transcriptional level. Furthermore, HIF activity is necessary for increased exon inclusion and the transcription of linear PDHK1 [69]. Mutational inactivation of VHL is considered the initial genetic event in most ccRCC cases, which leads to the accumulation of HIF transcription factors. HIF-2A has been generally implicated as an oncoprotein, whereas HIF-1A is an inhibitor of ccRCC tumorigenesis and development [22]. HIF-2A is a major player in ccRCC pathogenesis and represents a potential therapeutic target [70]. We investigated whether HIF-2A upregulates circPDHK1 expression by interacting with the genomic PDHK1 promoter. ChIP assays indicated specific interactive regions of HIF-2A at the PDHK1 promoter, and Pearson’s correlation analysis confirmed the association between circPDHK1 and HIF-2A expression in ccRCC tissue samples. Our study provides new insights into the mechanism underlying circPDHK1 generation and the etiology of ccRCC.

## Conclusions

In the present study, we found that circPDHK1 was highly expressed in ccRCC tissues and was positively associated with ccRCC tumor stage and grade. CircPDHK1 encodes a novel peptide, PDHK1-241aa, that is translated via its endogenous IRES. Moreover, knockdown and overexpression of circPDHK1 or mutation of its initiation codon verified that this novel peptide promotes ccRCC proliferation and metastasis. Additionally, HIF-2A may upregulate circPDHK1 by activating pre-PDHK1 RNA transcription, thereby facilitating PDHK1-241aa translation. Furthermore, our results revealed that PDHK1-241aa interacted with PPP1CA, leading to its translocation into the nucleus, which inhibited AKT dephosphorylation and activated the AKT-mTOR signaling pathway (Fig. 8H). Our study provides new insights into the multiplicity of circRNAs and the mechanisms underlying circRNA translation products. Moreover, these findings highlight the potential utility of circPDHK1 as a biomarker and therapeutic target in ccRCC.

### Supplementary Information

**Additional file 1: Table S1.** Association between clinicopathological characteristic and circPDHK1 expression in ccRCC patients in validation phase. **Table S2.** Clinicopathological features of 4 patients with ccRCC in circRNA microarray. **Table S3.** Information of shRNAs and siRNAs. **Table S4.** Information of RNA probes. **Table S5.** Sequences of primers. **Table S6.** The differentially expressed circRNAs in ccRCC tissues compared to matched noncancerous tissues with WGCNA analysis. **Table S7.** Candidate differentially-expressed circRNAs. **Table S8.** Candidate binding proteins detected by Flag-IP and LC-MS. **Table S9.** Association between clinicopathological characteristic and HIF-2A expression in ccRCC patients in validation phase.

**Additional file 2: Figure S1.** Screening and identification of upregulated circPDHK1 expression in ccRCC tissues and cells. (A) The volcano plot showing circRNAs expression in 4 ccRCC vs. 4 paired adjacent normal tissues from circRNA microarray. (B) Phylogenetic tree (Gene co-expression module analysis). horizontal coordinate: the gene co-expression modules analysis results show that different colors represent different gene modules. Vertical coordinate: the coefficient of difference between genes, each branch of the evolutionary tree represents a gene. (C) Trait related trait module analysis. Each column represents a trait, and each row represents a gene module. The numerical values in each cell indicate the correlation between the module and the trait, with values closer to 1 indicating a stronger positive correlation, and values closer to -1 indicating a stronger negative correlation. The numbers in parentheses represent the significance *P* value, where smaller values indicate stronger significance. The *P* values are calculated using the Student's t-test, with smaller *P* values indicating a stronger significance in the correlation between the trait and the module. (D) The volcano plot of differentially expressed circRNAs between ccRCC and adjacent tissues in GSE100186 (|log2(fold change)|>2, *p*<0.05). (E) The expression proportions of circPDHK1 in ccRCC paired tissues was detected by qRT-PCR (*n*=148). (F) RT‒qPCR was used to analyze the relative expression of circPDHK1 in the indicated cell lines. *P < 0.05; **P < 0.01.** Figure S2.** Inhibition of circPDHK1 suppresses the proliferation of ccRCC cells *in vivo*. (A-B) RT-qPCR measurements of knockdown efficiency of two different circPDHK1 siRNAs on circPDHK1 and linearPDHK1 expression in Caki-1 and 786-O cells as indicated. (C) Volumes of xenograft Caki-1 tumors in NSG mice following injection of cholesterol-modified circPDHK1 siRNA. Tumor volumes were calculated every 2 d. At Day 21 after treatment, all mice were sacrificed, and (D) subcutaneous tumors were dissected and recorded. (E) Subcutaneous tumor was also photographed. (*n*=3, each group). The result with a trend but no significance. (F) RT-qPCR was detected knockdown efficiency of circPDHK1 in tumor tissues. (G-H) Representative images and quantification of Ki-67 immunohistochemical (IHC) staining of subcutaneous Caki-1 tumor sections. (I) RT-qPCR was used to detected knockdown efficiency of circPDHK1 following lentivirus infection in different MOI values. *P < 0.05; **P < 0.01; ***P < 0.001.** Figure S3.** CircPDHK1 encodes a novel peptide PDHK1-241aa. (A) Translation potential ability prediction of circPDHK1 on Transcirc database. (B) IRES sequence prediction of circPDHK1 on circRNADb. (C) Potential m6A modification sites predicted using SRAMP (https://www. cuilab.cn/sramp/). (D) RNA methylation of measured using the meRIP assay. (E) RT-qPCR measurements of overexpression efficiency in Caki-1 and 786-O cells. (F) The PDHK1-241aa peptide sequence encoded by circPDHK1 was authenticated by LC‒MS analysis. (G) Anti-PDHK1 antibody epitope recognition compared with the protein sequence of PDHK1-241aa and linear PDHK1. (H) Western blot detection of the expression level of PDHK1-241aa in 16 paired ccRCC samples and normal adjacent tissues. (I) Western blot was used to detected the relative expression levels of PDHK1-241aa in the indicated cell lines. **P < 0.01; ***P < 0.001; ns, no significance.** Figure S4.** Construction and efficiency verification of circPDHK1 overexpression vector. (A) Schematic illustration of the expression plasmids. Measurements of overexpression efficiency of the vector at the (B-C) RNA level using RT-qPCR and at the (D-E) protein level using western blot. ***P < 0.001.** Figure S5.** PDHK1-241aa promotes the proliferation, migration and invasion of ccRCC cells in vitro following transfection with circPDHK1 CDS vector. (A) Colony formation assay (B) CCK-8 assay and (C-D) EdU assay were performed to detect the cell viability and proliferation activity of Caki-1 and 786-O cells transfected with pCDH and circPDHK1 CDS overexpression vectors. Bars = 200 μm. (E-G) Transwell assay to detect cell migration and invasion and (H-I) wound healing assays were conducted to evaluate cell migration abilities in Caki-1 and 786-O cells tansfected with pCDH and circPDHK1 CDS vectors. Bars = 100 μm.*P < 0.05; **P < 0.01; ***P < 0.001.** Figure S6.** PDHK1-241aa promotes the proliferation of ccRCC cells *in vivo*. (A) Tumor volume following injection of circPDHK1 and circPDHK1 ATG mutant overexpression lentivirus. Volumes were calculated every 2 d. (B) At Day 17 after treatment, all mice were sacrificed, and tumor weights in each group as indicated. The result with a trend but no significance. (C) Images of subcutaneous Caki-1 tumors (*n*=5, each group). (D) RT-qPCR was detected overexpression efficiency of vectors in tumor tissues as indicated. (E-F) Representative images and quantification of Ki-67 IHC staining of tumors. ***P < 0.001; ns, no significance.** Figure S7.** The effect of PDHK1-241aa on the expression of relevant proteins within the AKT-mTOR signaling pathway. (A-B) Western blot detection of additional proteins (PI3K, p-PI3K, PDK1, PTEN) related to the AKT-mTOR pathway in Caki-1 and 786-O cells following circPDHK1 interference and overexpression as indicated. The antibodies used in the present study were as follows: rabbit anti-PI3K (CST, Cell Signaling Technology, Danvers, MA, USA #4257S, 1:1000 dilution), rabbit anti-p-PI3K (CST; Cell Signaling Technology, Danvers, MA, USA, #4288T, 1:1000 dilution), rabbit anti-PTEN (CST; Cell Signaling Technology, Danvers, MA, USA, #9559T, 1:1000 dilution), rabbit anti-PDK1 (Proteintech, Wuhan, China, 17086-1-AP, 1:1000 dilution), rabbit anti-PDHK1/PDHK1-241aa (Novus, Centennial, CO, USA, NBP1-85955, 1:1000 dilution), anti-β-actin (ZSGB-BIO, China, ZM-0001, 1:1000 dilution).** Figure S8.** Small molecule inhibitors significantly counteracted circPDHK1-overexpression-induced enhancement of cell viability activity in ccRCC cells. (A) The effect of treatment with small molecule inhibitors (PT-2385: HIF-2A inhibitor, LY-294002: PI3K inhibitor, Rapamycin: mTOR inhibitor) at different concentrations in Caki-1 and 786-O cells. (B) RT-qPCR was detected the relative expression of circPDHK1 after two days of treatment with small molecule inhibitors in Caki-1 and 786-O cells. (C) CCK-8 rescue assay was performed to detect the cell viability activity after treatment with small molecule inhibitors in Caki-1 and 786-O cells transfected with pLC5-ciR or circPDHK1 overexpression vector. *P < 0.05; **P < 0.01; ***P < 0.001; ns, no significance.** Figure S9.** Small molecule inhibitors significantly counteracted circPDHK1-overexpression-induced enhancement of proliferation and migration in ccRCC cells. (A) EdU rescue assay was performed to detect the proliferation activity after two days treatment with small molecule inhibitors in Caki-1 and 786-O cells transfected with pLC5-ciR or circPDHK1 overexpression vector. Representative images and quantification as shown. Bars = 200 μm. (B) Transwell rescue assay was performed to detect the migration activity after two days treatment with small molecule inhibitors in Caki-1 and 786-O cells transfected with pLC5-ciR or circPDHK1 overexpression vector. Representative images and quantification as shown. Bars = 100 μm. *P < 0.05; **P < 0.01; ***P < 0.001; ns, no significance.** Figure S10.** PDHK1-241aa interacted with PPP1CA. (A-B) RT-qPCR and western blot detected the regulation of circPDHK1 and PPP1CA overexpression or interference on each other's expression level. (C) GEPIA-2 database was used to analyze PPP1CA mRNA expression level in ccRCC. (D) RT-qPCR analysis of PPP1CA expression in additional 24 paired ccRCC samples and normal adjacent tissues. (E) Western blot analysis of circPDHK1 expression in 8 paired ccRCC samples and normal adjacent tissues. (F) Immunofluorescence assay was used to verify the localization of PPP1CA in Caki-1 and 786-O wild cells. The red (anti-PPP1CA) indicated the PPP1CA; The blue (hochest) indicated the nucleus. Bars = 20 μm. (G) We also established truncation mutants BCDHK domain 3×Flag and HATPase domain 3×Flag plasmid. Co-IP assay was used to verify the binding site of PPP1CA and circPDHK1. Immunoblot analysis following cotransfection with PPP1CA and the indicated truncation mutants. ImageJ software was used to quantify gray values. ns, no significance.** Figure S11.** The expression of circPDHK1 or PPP1CA in rescue experiments. (A-B) RT-qPCR was detected the knockdown and overexpression efficiency of circPDHK1 and PPP1CA in rescue experiments. *P < 0.05; **P < 0.01; ***P < 0.001; ns, no significance.** Figure S12.** Phenotypes of Caki-1 and 786-O cells with co-overexpression of circPDHK1 and PPP1CA. (A) Colony formation assay (B) CCK-8 assay and (C-D) EdU assay were performed to detect the cell viability and proliferation activity of Caki-1 and 786-O cells transfected with circPDHK1 or/and PPP1CA overexpression vectors. Bars = 200 μm. (E-G) Transwell migration and invasion assays to detect cell migration ability of Caki-1 and 786-O cells cotransfected with the pLC5-ciR and pCDH vector, a circPDHK1 overexpressed vector and pCDH vector, the pLC5-ciR and PPP1CA overexpressed vector, circPDHK1 overexpressed vector and the PPP1CA overexpressed vector. Bars = 100 μm. (H) Western blot detection of the phosphorylation levels of AKT-mTOR signaling pathway. (I) RT-qPCR analysis to detect the cholesterol-modified siRNA efficiency in mouse tumors. *P < 0.05; **P < 0.01; ***P < 0.001; ns, no significance.** Figure S13.** Impact of circPDHK1 knockdown on the sensitivity of ccRCC to small-molecule targeted drugs. (A) CCK-8 was used to detect IC50 values of circPDHK1 knockdown Caki-1 and 786-O cells treated with small molecule inhibitors. (Sunitinib: a multi-targeted RTK inhibitor, Pazopanib: a multi-targeted tyrosine kinase inhibitors) (B) CCK-8 rescue assay was performed to detect the cell viability activity after two days treatment with small molecule inhibitors in Caki-1 and 786-O cells transfected with control siRNA or circPDHK1 siRNA. ns, no significance. *P < 0.05; **P < 0.01; ***P < 0.001; ns, no significance.** Figure S14.** The effect of circPDHK1 knockdown with small molecule inhibitors on the proliferation of Caki-1 and 786-O cells. EdU assay was performed to detect the proliferation activity after two days treatment with small molecule inhibitors in Caki-1 and 786-O cells transfected with control siRNA or circPDHK1 siRNA. Representative images and quantification as shown. Bars = 200 μm. *P < 0.05; **P < 0.01; ***P < 0.001; ns, no significance.** Figure S15.** The effects of circPDHK1 knockdown with small molecule inhibitors on the migration of Caki-1 and 786-O cells. Transwell assay was performed to detect the migration activity after two days treatment with small molecule inhibitors in Caki-1 and 786-O cells transfected with control siRNA or circPDHK1 siRNA. Representative images and quantification as shown. Bars = 100 μm. ns, no significance. *P < 0.05; **P < 0.01; ***P < 0.001; ns, no significance.** Figure S16.** Relative expression of HIF-1A in ccRCC and the expression of circPDHK1 or HIF-2A after silencing circPDHK1 or overexpressing HIF-2A in the rescue experiment. (A) Relative expression of HIF-1A taken from data in the TCGA database (http://gepia2.cancer-pku.cn/). (B) RT-qPCR analysis to detected the effect of si-circPDHK1, HIF-2A overexpression, and si-circPDHK1 + OE-HIF-2A on the expression of circPDHK1 or HIF-2A in rescue experiments. **P < 0.01; ***P < 0.001; ns, no significance.

## Data Availability

All data generated or analyzed in this study are included in this article or in the supplementary material. Original data supporting the results of this study are available from the corresponding author upon reasonable request.

## Abbreviations

ccRCC: Clear cell renal cell carcinoma
circRNA: Circular RNA
FISH: Fluorescent in situ hybridization
RIP: RNA immunoprecipitation
IF: Immunofluorescence
CCK-8: Cell counting kit-8
ActD: Actinomycin D
EdU: 5-Ethynyl-2'-deoxyuridine
Co-IP: Coimmunoprecipitation
IP-MS: Immunoprecipitation-mass spectrometry
ChIP: Chromatin immunoprecipitation
IHC: Immunohistochemistry
HE: Hematoxylin–eosin
IRES: Internal ribosomal entry sites
m6A: N6-methyladenosine
TKI: Tyrosine kinase inhibitors
WHO/ISUP: World Health Organization / International Society of Urological Pathology
