# Supplementary material for: A novel peptide PDHK1-241aa encoded by circPDHK1 promotes ccRCC progression via interacting with PPP1CA to inhibit AKT dephosphorylation and activate the AKT-mTOR signaling pathway
Source: Mol Cancer. 2024 Feb 15;23:34. doi: 10.1186/s12943-024-01940-0 (PMC10870583; doi:10.1186/s12943-024-01940-0)
Supplement: Supplementary file 2 — Additional file 2: Figure S1. Screening and identification of upregulated circPDHK1 expression in ccRCC tissues and cells. (A) The volcano plot showing circRNAs expression in 4 ccRCC vs. 4 paired adjacent normal tissues from circRNA microarray. (B) Phylogenetic tree (Gene co-expression module analysis). horizontal coordinate: the gene co-expression modules analysis results show that different colors represent different gene modules. Vertical coordinate: the coefficient of difference between genes, each branch of the evolutionary tree represents a gene. (C) Trait related trait module analysis. Each column represents a trait, and each row represents a gene module. The numerical values in each cell indicate the correlation between the module and the trait, with values closer to 1 indicating a stronger positive correlation, and values closer to -1 indicating a stronger negative correlation. The numbers in parentheses represent the significance P value, where smaller values indicate stronger significance. The P values are calculated using the Student's t-test, with smaller P values indicating a stronger significance in the correlation between the trait and the module. (D) The volcano plot of differentially expressed circRNAs between ccRCC and adjacent tissues in GSE100186 (|log2(fold change)|>2, p<0.05). (E) The expression proportions of circPDHK1 in ccRCC paired tissues was detected by qRT-PCR (n=148). (F) RT‒qPCR was used to analyze the relative expression of circPDHK1 in the indicated cell lines. *P < 0.05; **P < 0.01. Figure S2. Inhibition of circPDHK1 suppresses the proliferation of ccRCC cells in vivo. (A-B) RT-qPCR measurements of knockdown efficiency of two different circPDHK1 siRNAs on circPDHK1 and linearPDHK1 expression in Caki-1 and 786-O cells as indicated. (C) Volumes of xenograft Caki-1 tumors in NSG mice following injection of cholesterol-modified circPDHK1 siRNA. Tumor volumes were calculated every 2 d. At Day 21 after treatment, all mice were sacrificed [file 12943_2024_1940_MOESM2_ESM.docx]

**
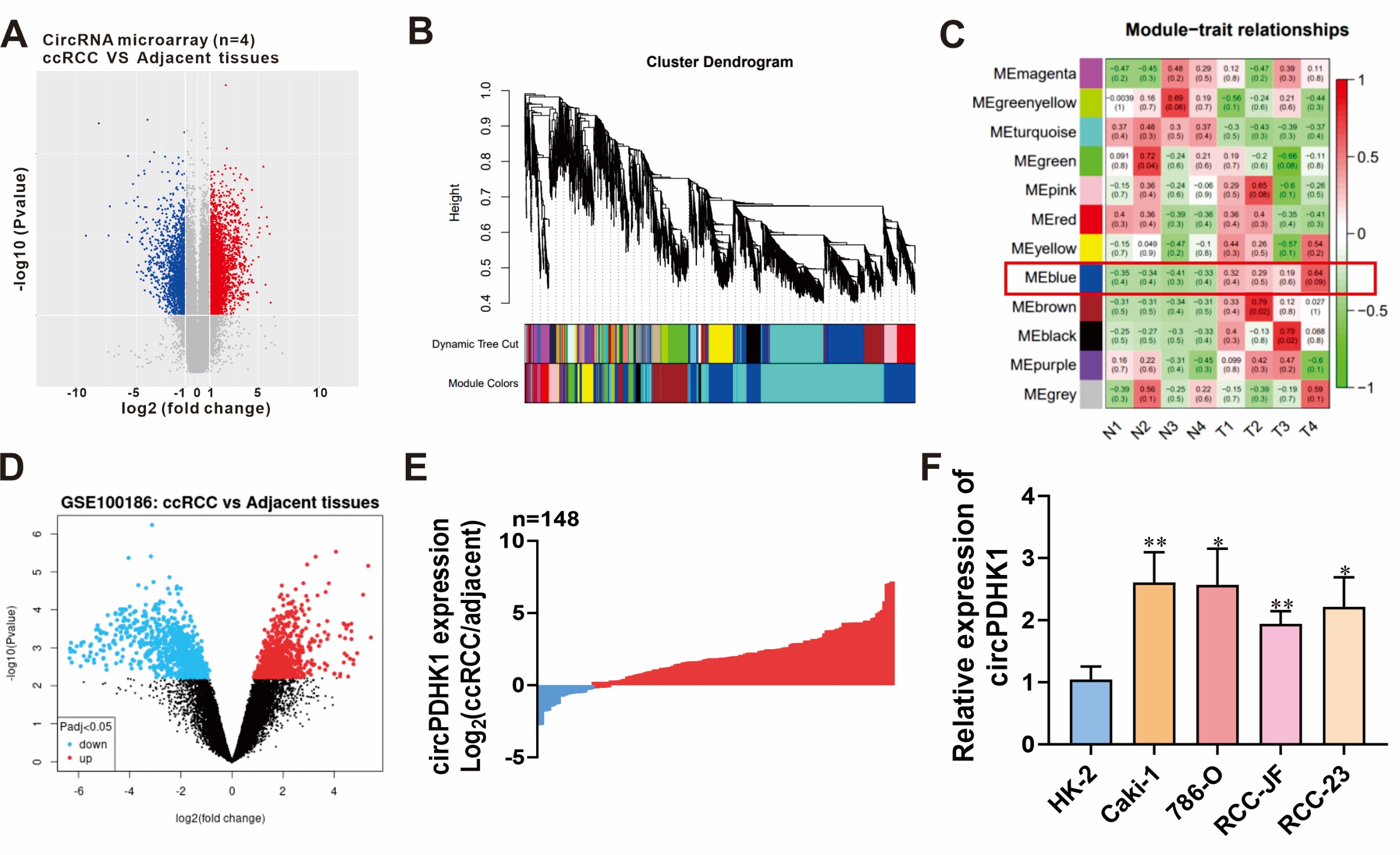
**

**Figure S1. Screening and identification of upregulated circPDHK1 expression in ccRCC tissues and cells. (A)** The volcano plot showing circRNAs expression in 4 ccRCC vs. 4 paired adjacent normal tissues from circRNA microarray. **(B)** Phylogenetic tree (Gene co-expression module analysis). horizontal coordinate: the gene co-expression modules analysis results show that different colors represent different gene modules. Vertical coordinate: the coefficient of difference between genes, each branch of the evolutionary tree represents a gene. **(C)** Trait related trait module analysis. Each column represents a trait, and each row represents a gene module. The numerical values in each cell indicate the correlation between the module and the trait, with values closer to 1 indicating a stronger positive correlation, and values closer to -1 indicating a stronger negative correlation. The numbers in parentheses represent the significance P value, where smaller values indicate stronger significance. The P values are calculated using the Student's t-test, with smaller P values indicating a stronger significance in the correlation between the trait and the module. **(D)** The volcano plot of differentially expressed circRNAs between ccRCC and adjacent tissues in GSE100186 (|log2(fold change)|>2, p<0.05). **(E)** The expression proportions of circPDHK1 in ccRCC paired tissues was detected by qRT-PCR (n=148). **(F)** RT‒qPCR was used to analyze the relative expression of circPDHK1 in the indicated cell lines. **P* < 0.05; ***P* < 0.01.


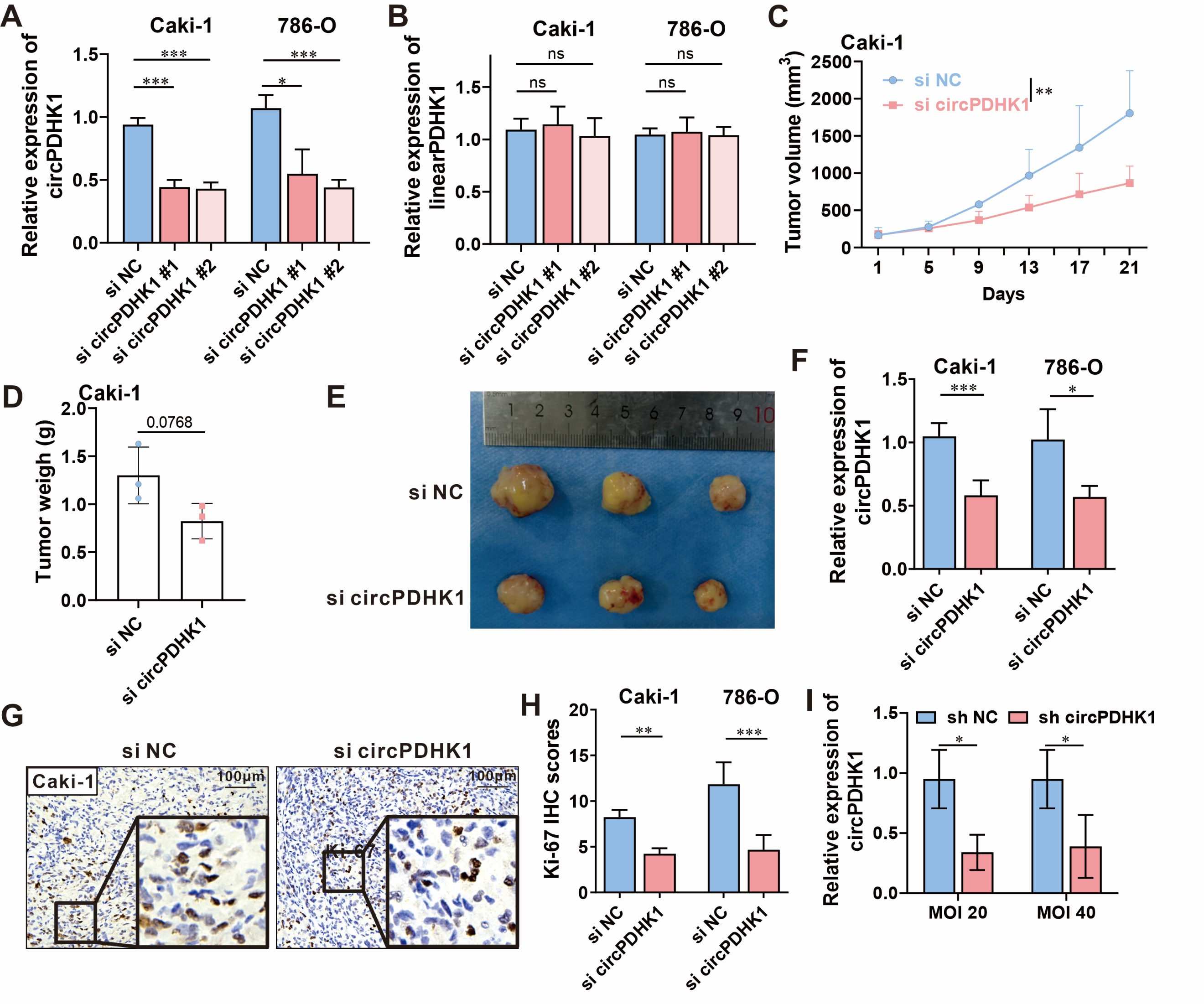


**Figure S2. Inhibition of circPDHK1 suppresses the proliferation of ccRCC cells *in vivo*. (A-B)** RT-qPCR measurements of knockdown efficiency of two different circPDHK1 siRNAs on circPDHK1 and linearPDHK1 expression in Caki-1 and 786-O cells as indicated. **(C)** Volumes of xenograft Caki-1 tumors in NSG mice following injection of cholesterol-modified circPDHK1 siRNA. Tumor volumes were calculated every 2 d. At Day 21 after treatment, all mice were sacrificed, and **(D)** subcutaneous tumors were dissected and recorded. **(E)** Subcutaneous tumor was also photographed. (n=3, each group). The result with a trend but no significance. **(F)** RT-qPCR was detected knockdown efficiency of circPDHK1 in tumor tissues. **(G-H)** Representative images and quantification of Ki-67 immunohistochemical (IHC) staining of subcutaneous Caki-1 tumor sections. **(I)** RT-qPCR was used to detected knockdown efficiency of circPDHK1 following lentivirus infection in different MOI values. **P* < 0.05; ***P* < 0.01; ****P* < 0.001.


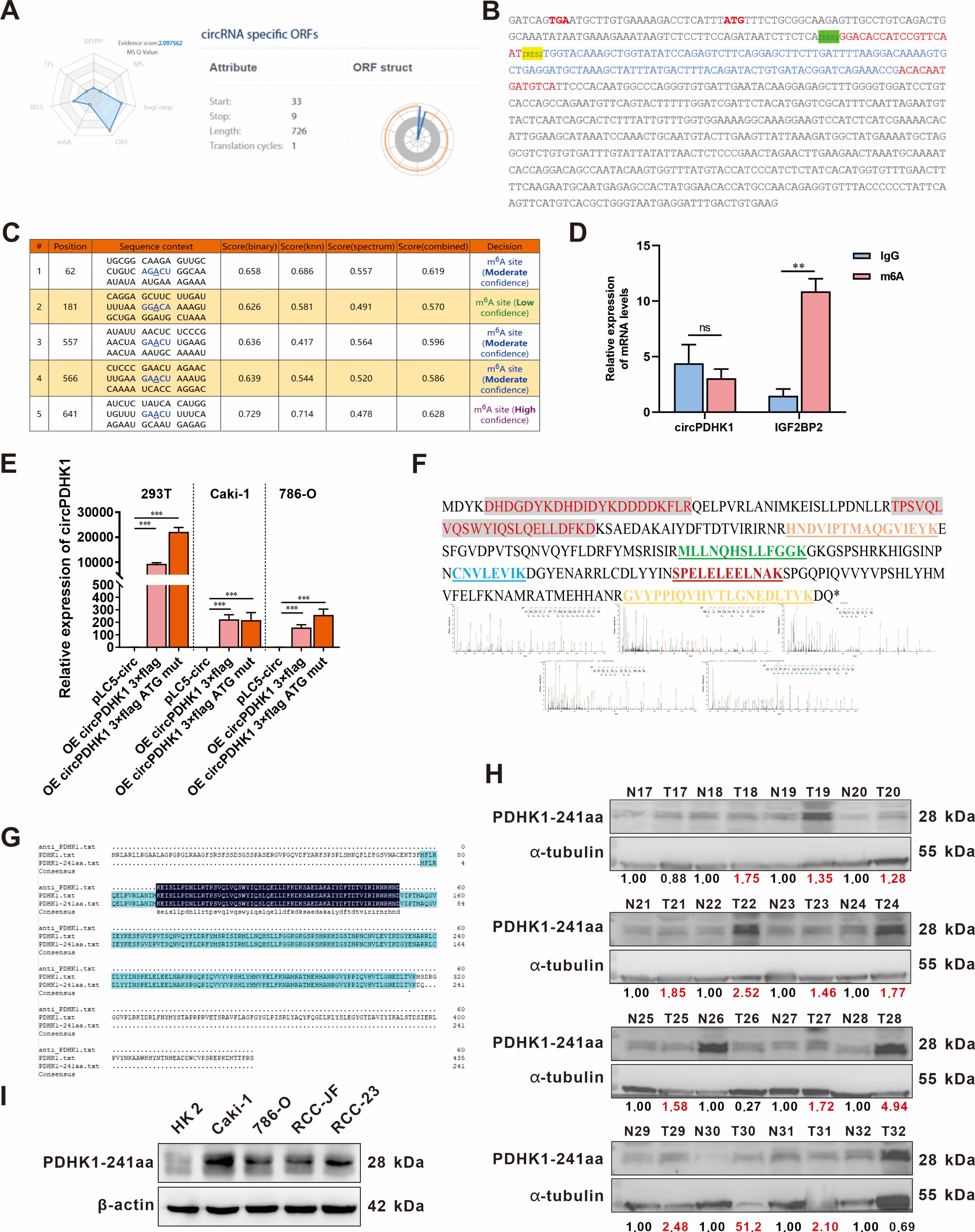


**Figure S3. CircPDHK1 encodes a novel peptide PDHK1-241aa. (A)** Translation potential ability prediction of circPDHK1 on Transcirc database. **(B)** IRES sequence prediction of circPDHK1 on circRNADb. **(C)** Potential m6A modification sites predicted using SRAMP (https://www. cuilab.cn/sramp/). **(D)** RNA methylation of measured using the meRIP assay. **(E)** RT-qPCR measurements of overexpression efficiency in Caki-1 and 786-O cells. **(F)** The PDHK1-241aa peptide sequence encoded by circPDHK1 was authenticated by LC‒MS analysis. **(G)** Anti-PDHK1 antibody epitope recognition compared with the protein sequence of PDHK1-241aa and linear PDHK1. **(H)** Western blot detection of the expression level of PDHK1-241aa in 16 paired ccRCC samples and normal adjacent tissues. **(I)** Western blot was used to detected the relative expression levels of PDHK1-241aa in the indicated cell lines. ***P* < 0.01; ****P* < 0.001; ns, no significance.


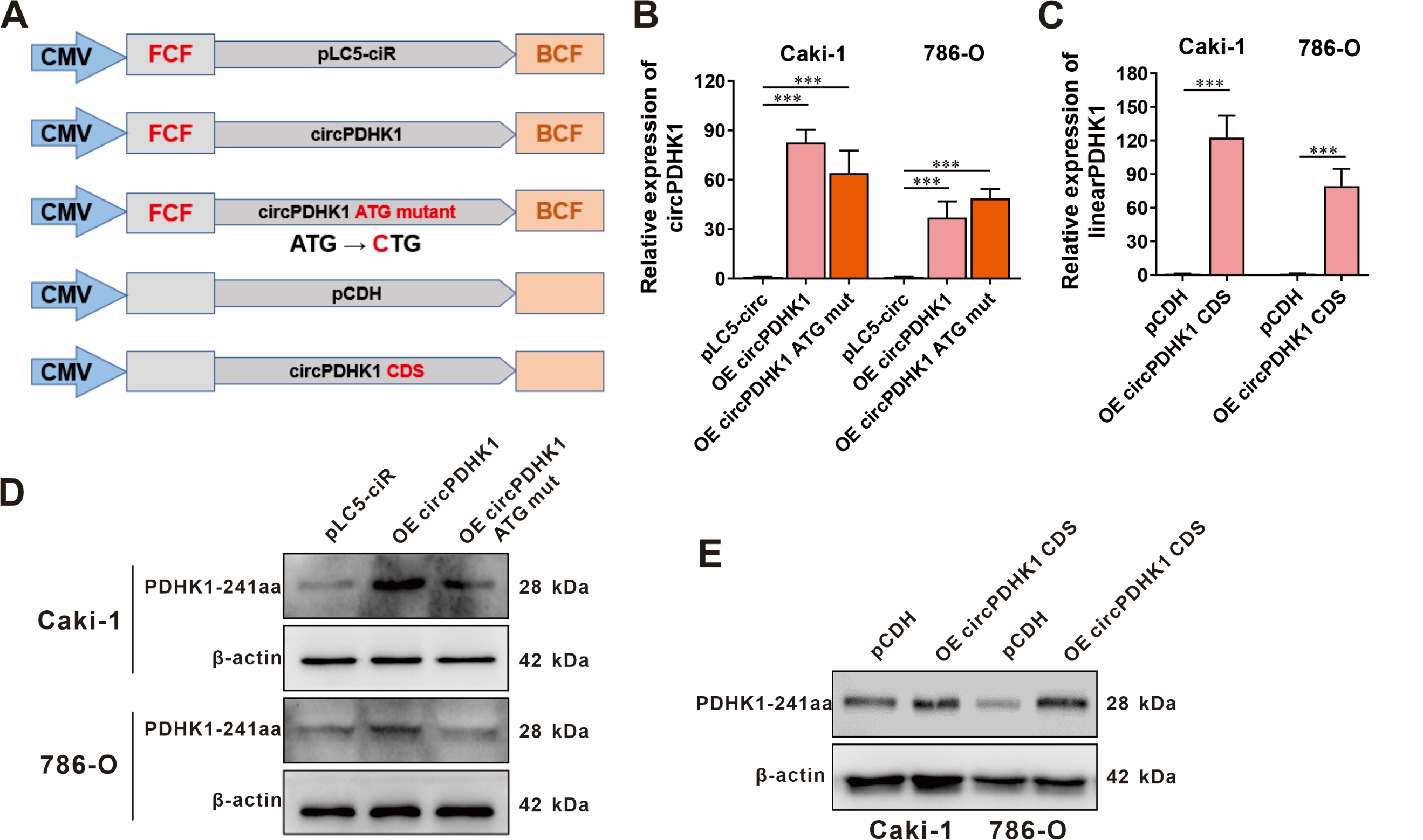


**Figure S4. Construction and efficiency verification of circPDHK1 overexpression vector. (A)** Schematic illustration of the expression plasmids. Measurements of overexpression efficiency of the vector at the **(B-C)** RNA level using RT-qPCR and at the **(D-E)** protein level using western blot. ****P* < 0.001.


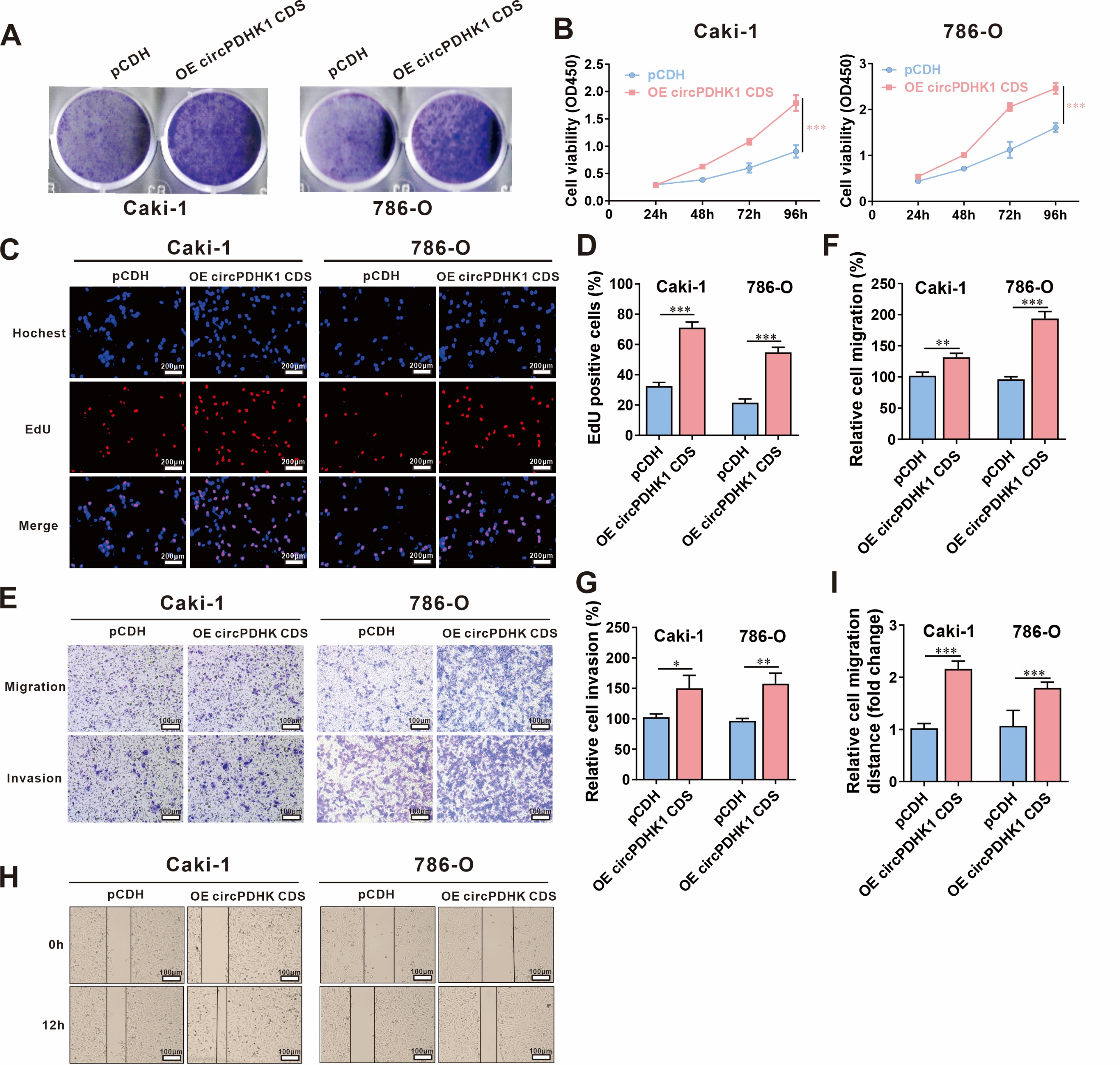


**Figure S5. PDHK1-241aa promotes the proliferation, migration and invasion of ccRCC cells in vitro** **following transfection with circPDHK1 CDS vector. (A)** Colony formation assay **(B)** CCK-8 assay and **(C-D)** EdU assay were performed to detect the cell viability and proliferation activity of Caki-1 and 786-O cells transfected with pCDH and circPDHK1 CDS overexpression vectors. Bars = 200 μm. **(E-G)** Transwell assay to detect cell migration and invasion and **(H-I)** wound healing assays were conducted to evaluate cell migration abilities in Caki-1 and 786-O cells tansfected with pCDH and circPDHK1 CDS vectors. Bars = 100 μm.**P* < 0.05; ***P* < 0.01; ****P* < 0.001.


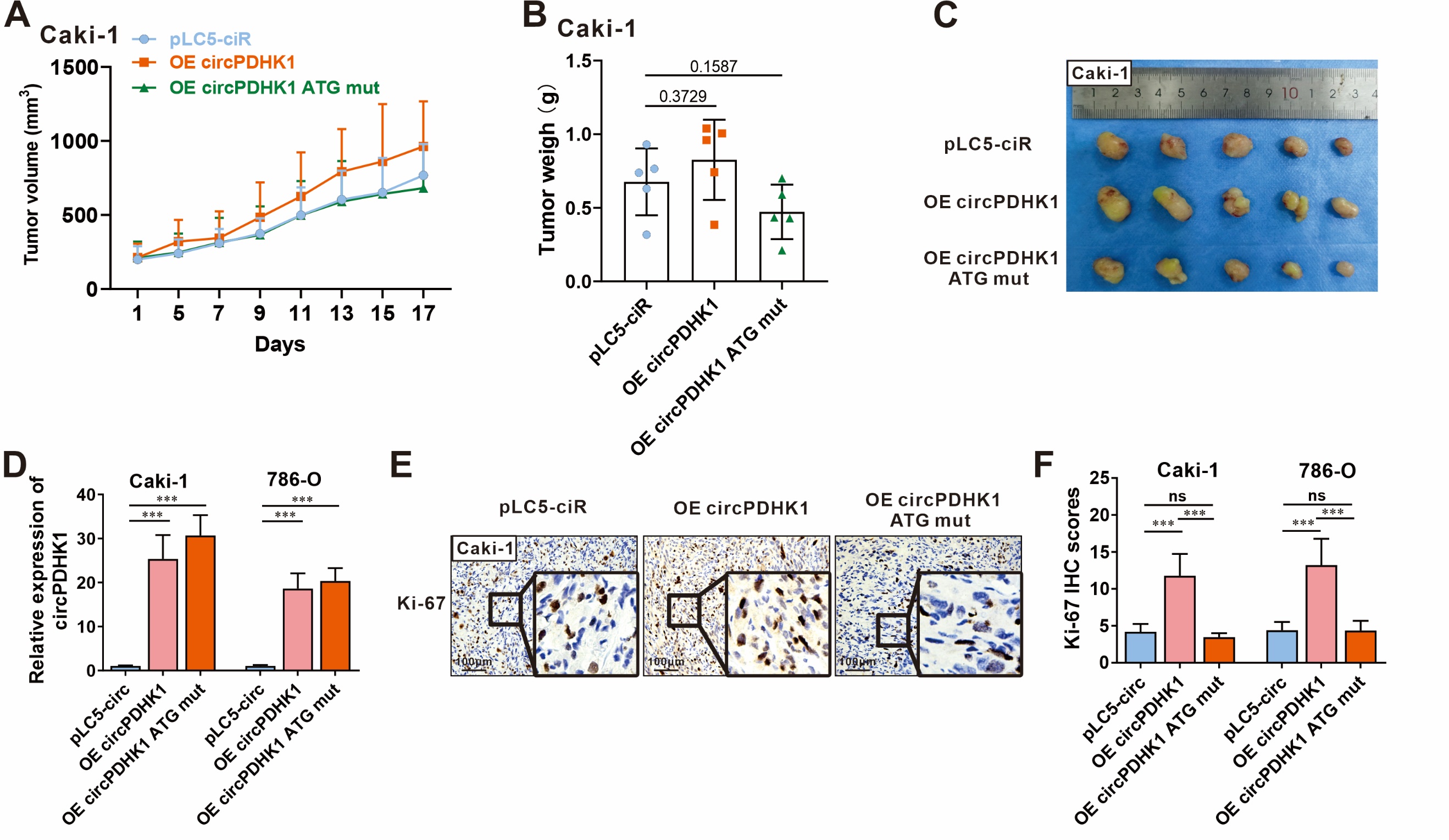


**Figure S6. PDHK1-241aa promotes the proliferation of ccRCC cells *in vivo*. (A)** Tumor volume following injection of circPDHK1 and circPDHK1 ATG mutant overexpression lentivirus. Volumes were calculated every 2 d. **(B)** At Day 17 after treatment, all mice were sacrificed, and tumor weights in each group as indicated. The result with a trend but no significance. **(C)** Images of subcutaneous Caki-1 tumors (n=5, each group). **(D)** RT-qPCR was detected overexpression efficiency of vectors in tumor tissues as indicated. **(E-F)** Representative images and quantification of Ki-67 IHC staining of tumors. ****P* < 0.001; ns, no significance.


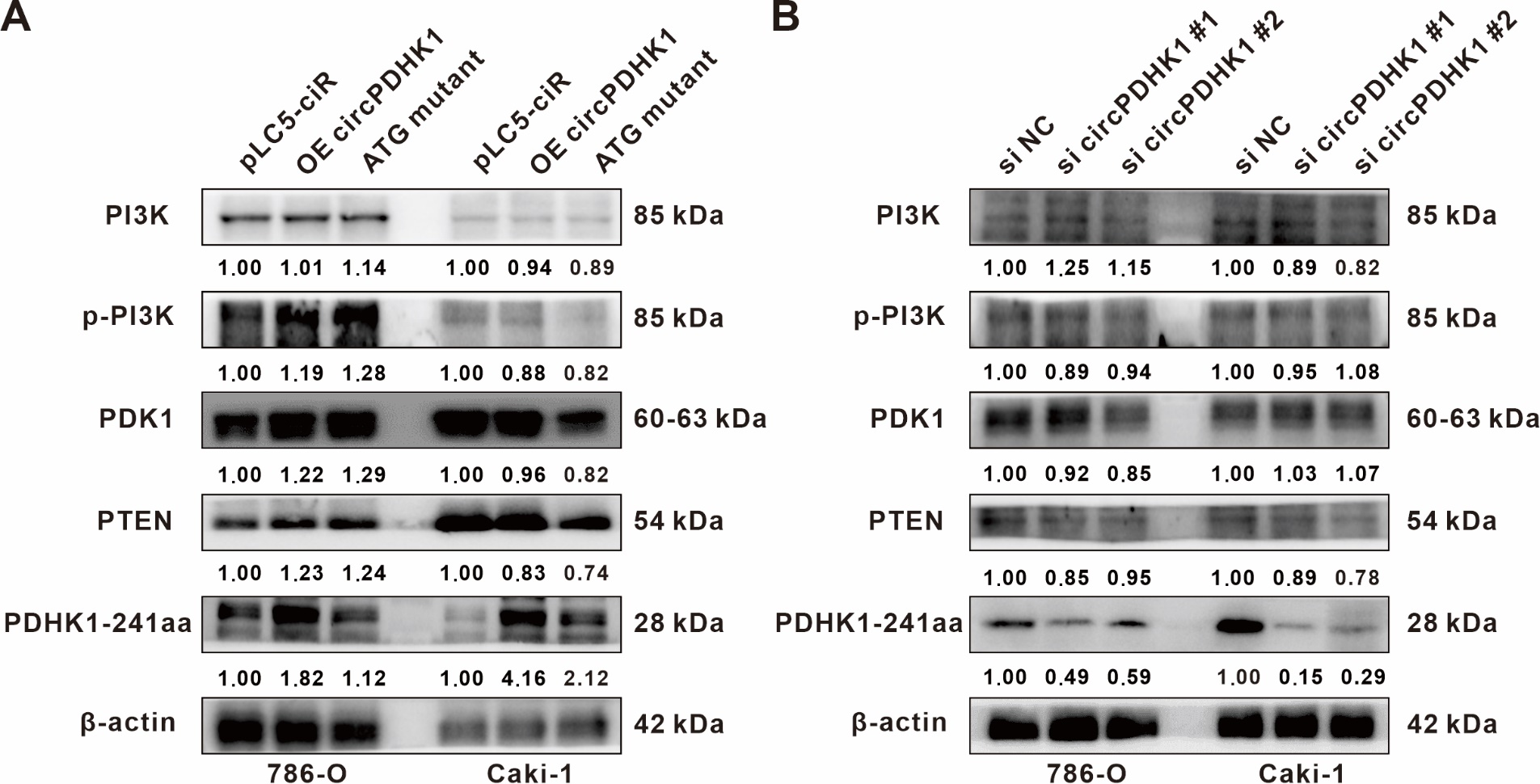


**Figure S7.** **The effect of PDHK1-241aa on the expression of relevant proteins within the AKT-mTOR signaling pathway. (A-B)** Western blot detection of additional proteins (PI3K, p-PI3K, PDK1, PTEN) related to the AKT-mTOR pathway in Caki-1 and 786-O cells following circPDHK1 interference and overexpression as indicated. The antibodies used in the present study were as follows: rabbit anti-PI3K (CST, Cell Signaling Technology, Danvers, MA, USA #4257S, 1:1000 dilution), rabbit anti-p-PI3K (CST; Cell Signaling Technology, Danvers, MA, USA, #4288T, 1:1000 dilution), rabbit anti-PTEN (CST; Cell Signaling Technology, Danvers, MA, USA, #9559T, 1:1000 dilution), rabbit anti-PDK1 (Proteintech, Wuhan, China, 17086-1-AP, 1:1000 dilution), rabbit anti-PDHK1/PDHK1-241aa (Novus, Centennial, CO, USA, NBP1-85955, 1:1000 dilution), anti-β-actin (ZSGB-BIO, China, ZM-0001, 1:1000 dilution).

**
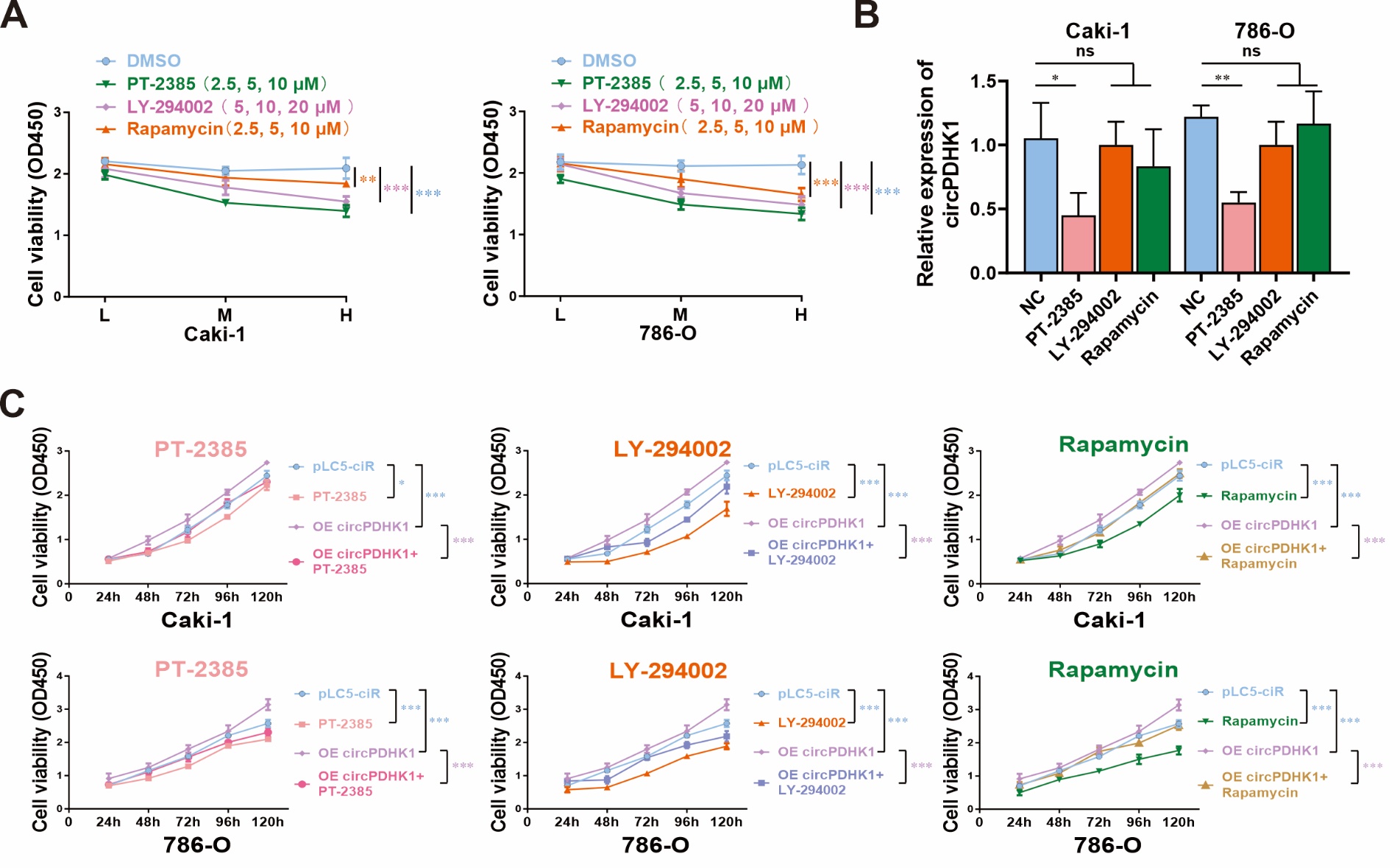
**

**Figure S8.** **Small molecule inhibitors significantly counteracted circPDHK1-overexpression-induced enhancement of cell viability activity in ccRCC cells. (A)** The effect of treatment with small molecule inhibitors (PT-2385: HIF-2A inhibitor, LY-294002: PI3K inhibitor, Rapamycin: mTOR inhibitor) at different concentrations in Caki-1 and 786-O cells. **(B)** RT-qPCR was detected the relative expression of circPDHK1 after two days of treatment with small molecule inhibitors in Caki-1 and 786-O cells. **(C)** CCK-8 rescue assay was performed to detect the cell viability activity after treatment with small molecule inhibitors in Caki-1 and 786-O cells transfected with pLC5-ciR or circPDHK1 overexpression vector. **P* < 0.05; ***P* < 0.01; ****P* < 0.001; ns, no significance.

**
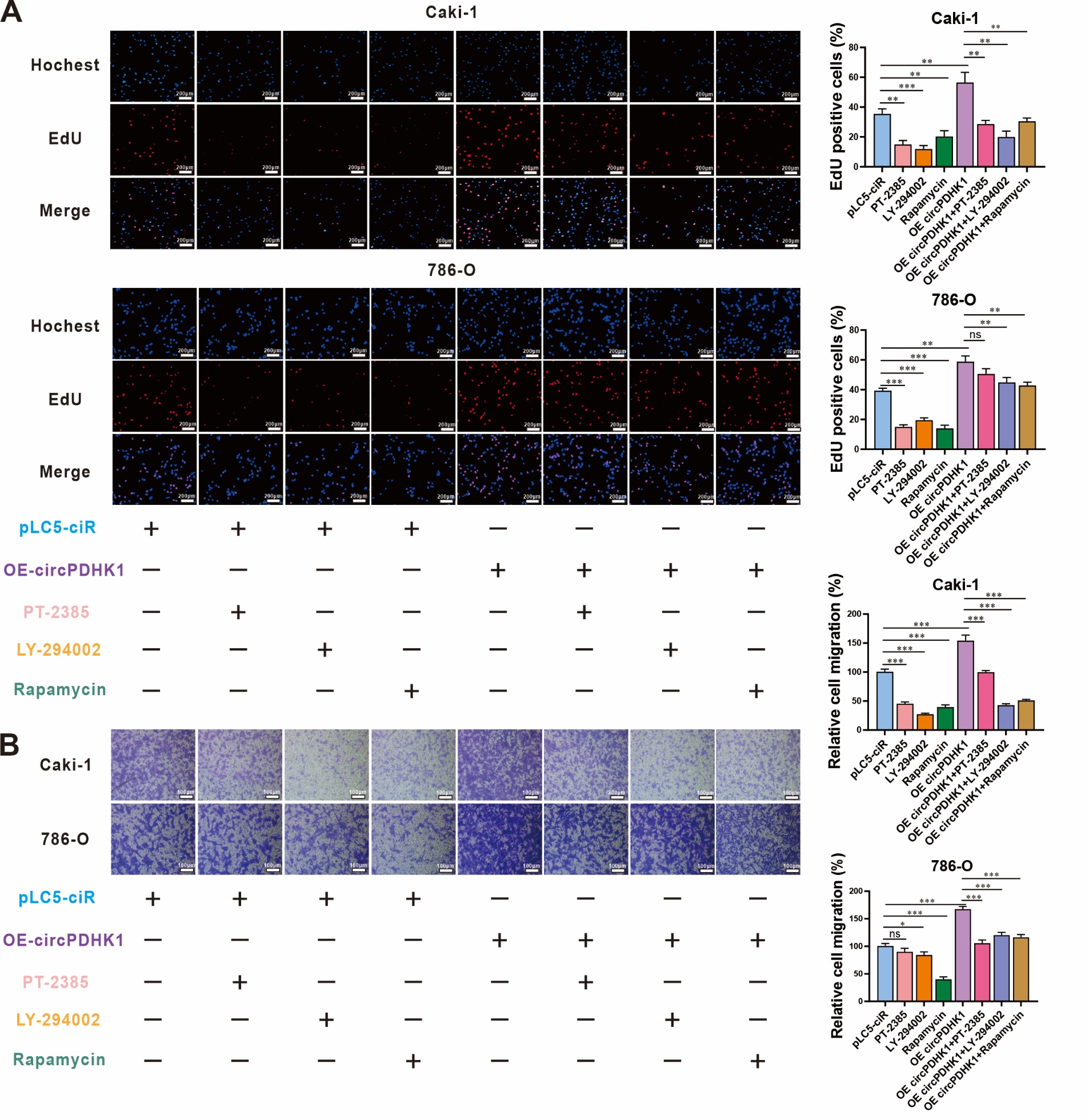
**

**Figure S9.** **Small molecule inhibitors significantly counteracted circPDHK1-overexpression-induced enhancement of proliferation and migration in ccRCC cells.** **(A)** EdU rescue assay was performed to detect the proliferation activity after two days treatment with small molecule inhibitors in Caki-1 and 786-O cells transfected with pLC5-ciR or circPDHK1 overexpression vector. Representative images and quantification as shown. Bars = 200 μm. **(B)** Transwell rescue assay was performed to detect the migration activity after two days treatment with small molecule inhibitors in Caki-1 and 786-O cells transfected with pLC5-ciR or circPDHK1 overexpression vector. Representative images and quantification as shown. Bars = 100 μm. **P* < 0.05; ***P* < 0.01; ****P* < 0.001; ns, no significance.

**
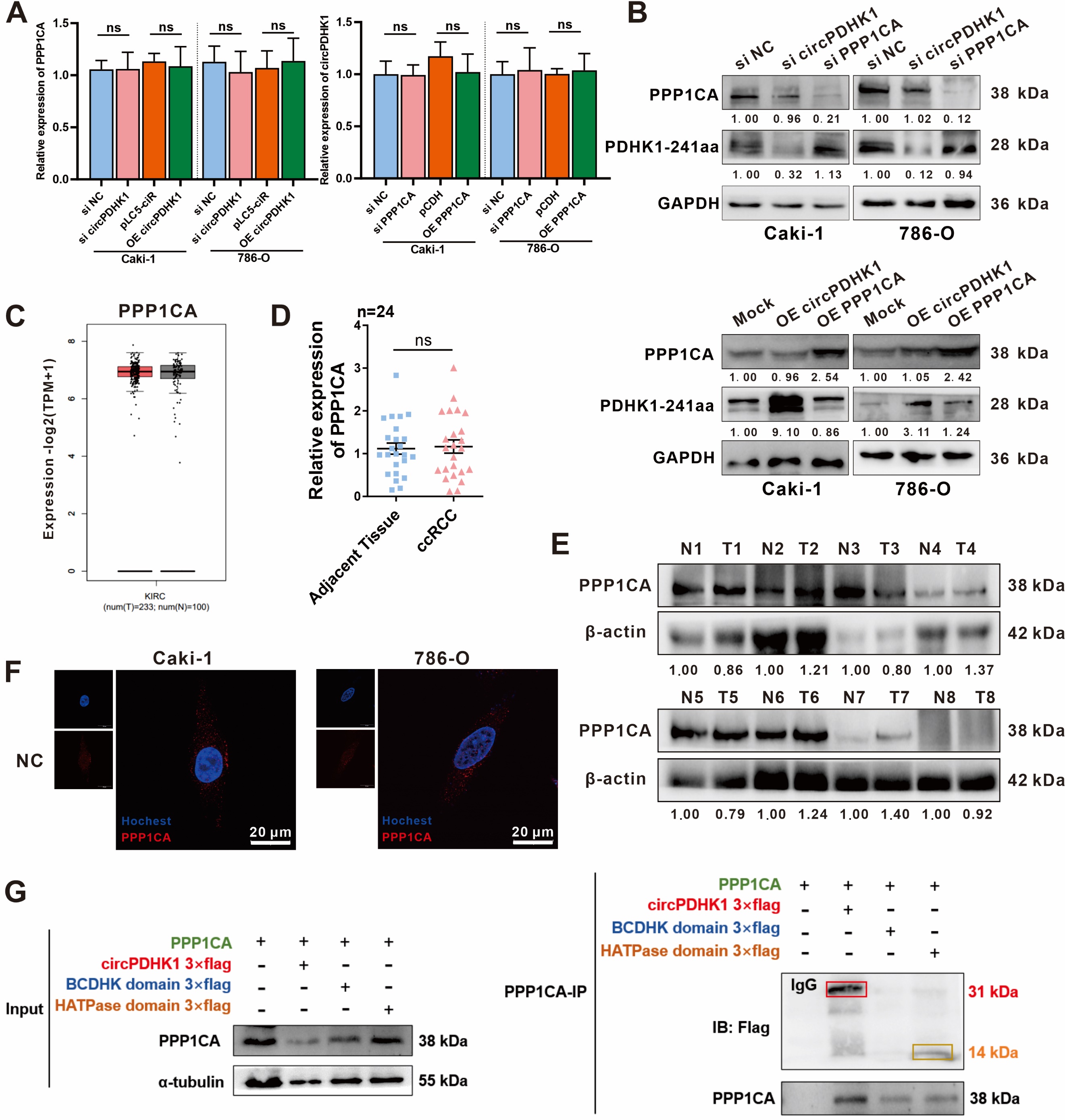
**

**Figure S10. PDHK1-241aa interacted with PPP1CA. (A-B)** RT-qPCR and western blot detected the regulation of circPDHK1 and PPP1CA overexpression or interference on each other's expression level. **(C)** GEPIA-2 database was used to analyze PPP1CA mRNA expression level in ccRCC. **(D)** RT-qPCR analysis of PPP1CA expression in additional 24 paired ccRCC samples and normal adjacent tissues. **(E)** Western blot analysis of circPDHK1 expression in 8 paired ccRCC samples and normal adjacent tissues. **(F)** Immunofluorescence assay was used to verify the localization of PPP1CA in Caki-1 and 786-O wild cells. The red (anti-PPP1CA) indicated the PPP1CA; The blue (hochest) indicated the nucleus. Bars = 20 μm. **(G)** We also established truncation mutants BCDHK domain 3×Flag and HATPase domain 3×Flag plasmid. Co-IP assay was used to verify the binding site of PPP1CA and circPDHK1. Immunoblot analysis following cotransfection with PPP1CA and the indicated truncation mutants. ImageJ software was used to quantify gray values. ns, no significance.
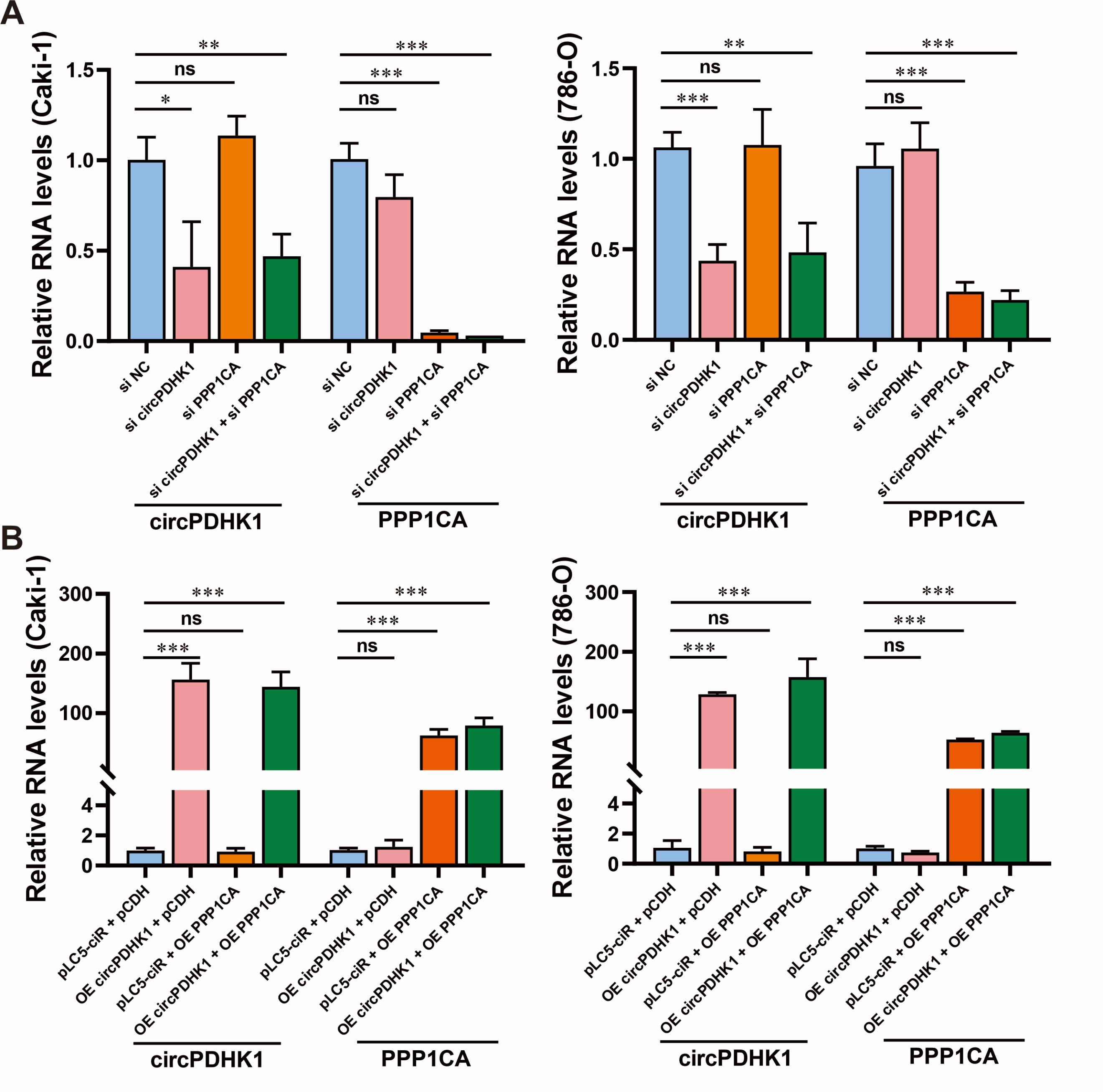


**Figure S11. The expression of circPDHK1 or PPP1CA in rescue experiments. (A-B)** RT-qPCR was detected the knockdown and overexpression efficiency of circPDHK1 and PPP1CA in rescue experiments. **P* < 0.05; ***P* < 0.01; ****P* < 0.001; ns, no significance.


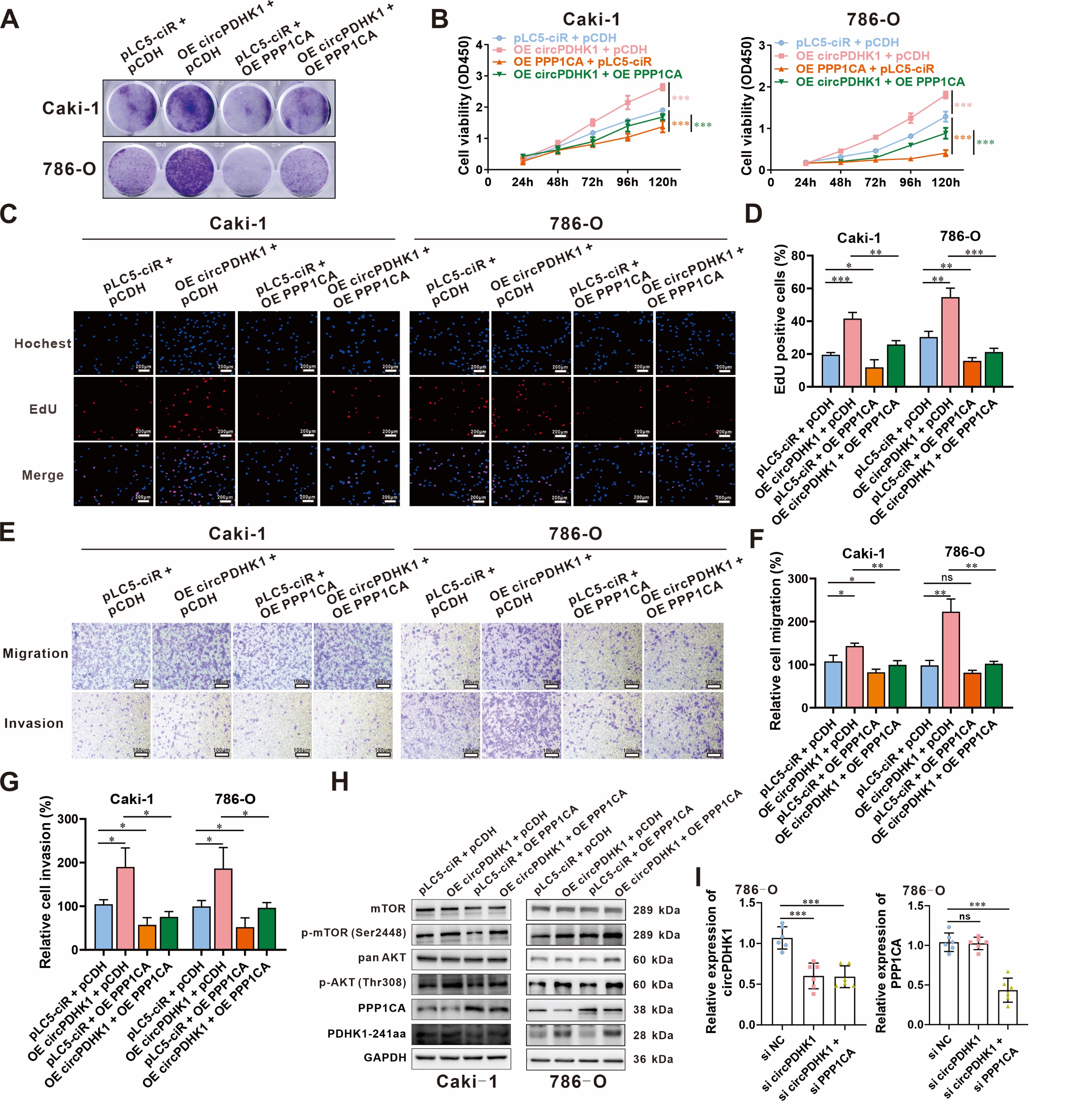


**Figure S12. Phenotypes of Caki-1 and 786-O cells with co-overexpression of circPDHK1 and PPP1CA. (A)** Colony formation assay **(B)** CCK-8 assay and **(C-D)** EdU assay were performed to detect the cell viability and proliferation activity of Caki-1 and 786-O cells transfected with circPDHK1 or/and PPP1CA overexpression vectors. Bars = 200 μm. **(E-G)** Transwell migration and invasion assays to detect cell migration ability of Caki-1 and 786-O cells cotransfected with the pLC5-ciR and pCDH vector, a circPDHK1 overexpressed vector and pCDH vector, the pLC5-ciR and PPP1CA overexpressed vector, circPDHK1 overexpressed vector and the PPP1CA overexpressed vector. Bars = 100 μm. **(H)** Western blot detection of the phosphorylation levels of AKT-mTOR signaling pathway. **(I)** RT-qPCR analysis to detect the cholesterol-modified siRNA efficiency in mouse tumors. **P* < 0.05; ***P* < 0.01; ****P* < 0.001; ns, no significance.

**
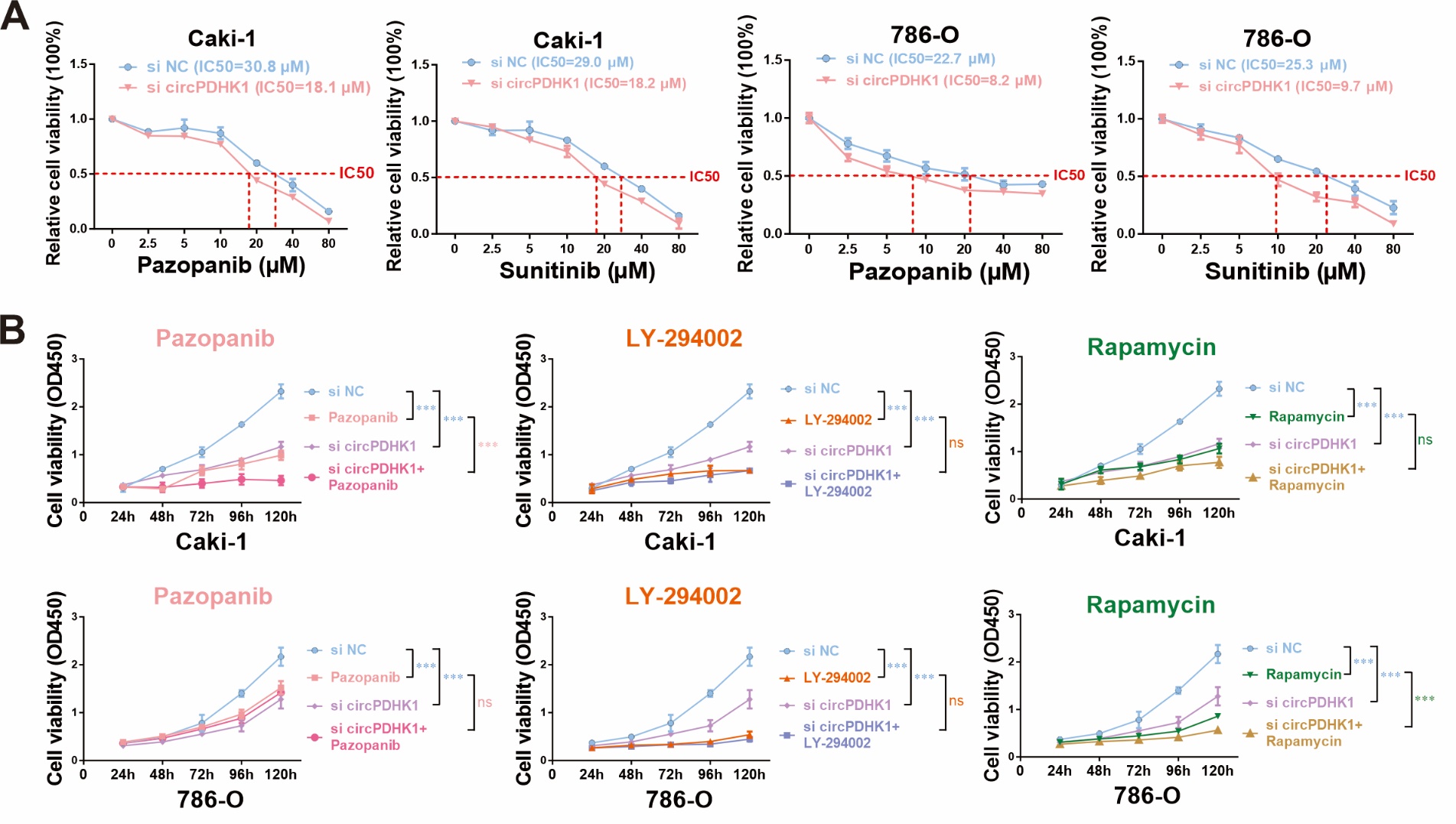
**

**Figure S13.** **Impact of circPDHK1 knockdown on the sensitivity of ccRCC to small-molecule targeted drugs. (A)** CCK-8 was used to detect IC50 values of circPDHK1 knockdown Caki-1 and 786-O cells treated with small molecule inhibitors. (Sunitinib: a multi-targeted RTK inhibitor, Pazopanib: a multi-targeted tyrosine kinase inhibitors) **(B)** CCK-8 rescue assay was performed to detect the cell viability activity after two days treatment with small molecule inhibitors in Caki-1 and 786-O cells transfected with control siRNA or circPDHK1 siRNA. ns, no significance. **P* < 0.05; ***P* < 0.01; ****P* < 0.001; ns, no significance.

**
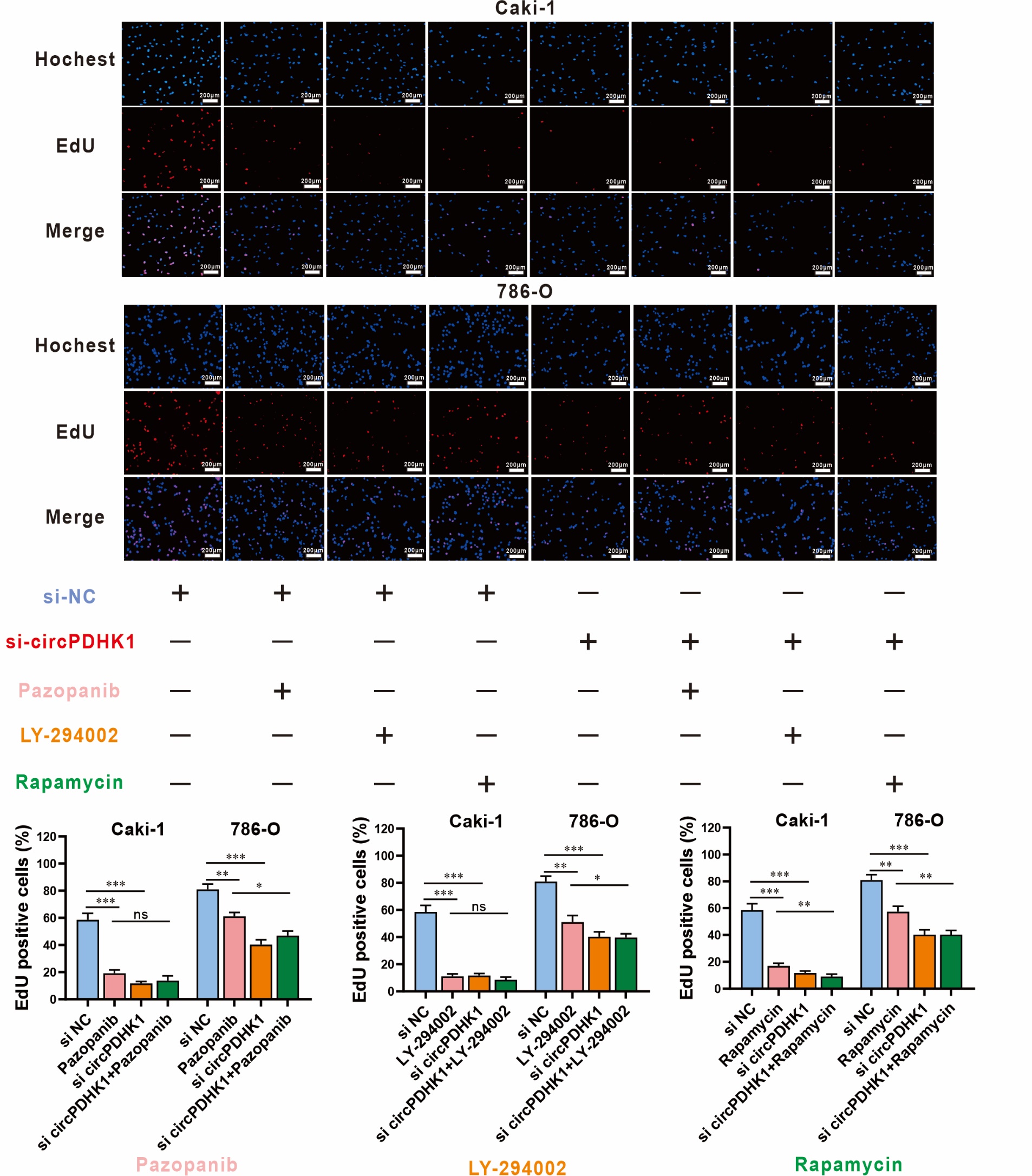
**

**Figure S14.** **The effect of circPDHK1 knockdown with small molecule inhibitors on the proliferation of Caki-1 and 786-O cells.** EdU assay was performed to detect the proliferation activity after two days treatment with small molecule inhibitors in Caki-1 and 786-O cells transfected with control siRNA or circPDHK1 siRNA. Representative images and quantification as shown. Bars = 200 μm. **P* < 0.05; ***P* < 0.01; ****P* < 0.001; ns, no significance.

**
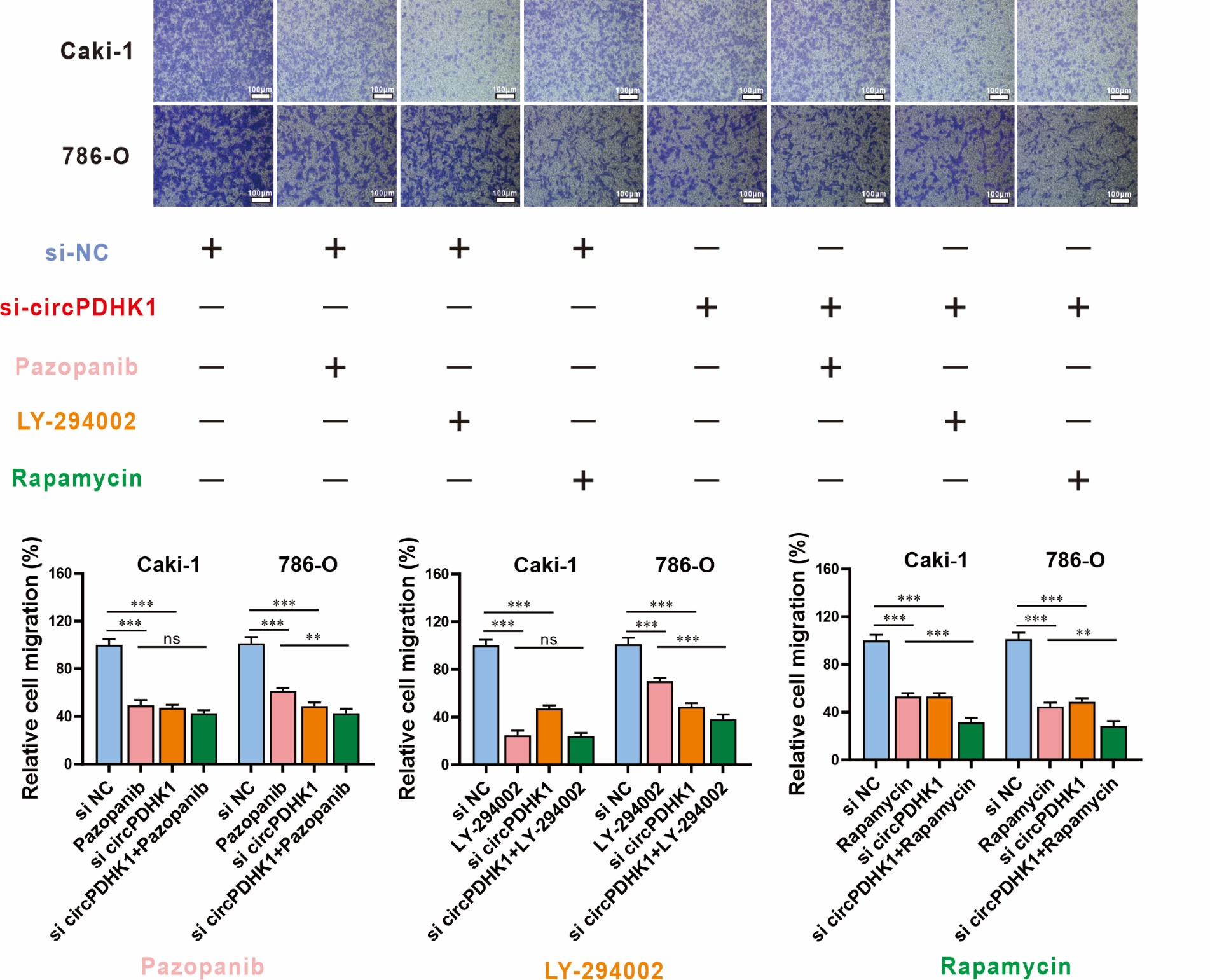
**

**Figure S15.** **The** **effects of circPDHK1 knockdown with small molecule inhibitors on the migration of Caki-1 and 786-O cells.** Transwell assay was performed to detect the migration activity after two days treatment with small molecule inhibitors in Caki-1 and 786-O cells transfected with control siRNA or circPDHK1 siRNA. Representative images and quantification as shown. Bars = 100 μm. ns, no significance. **P* < 0.05; ***P* < 0.01; ****P* < 0.001; ns, no significance.


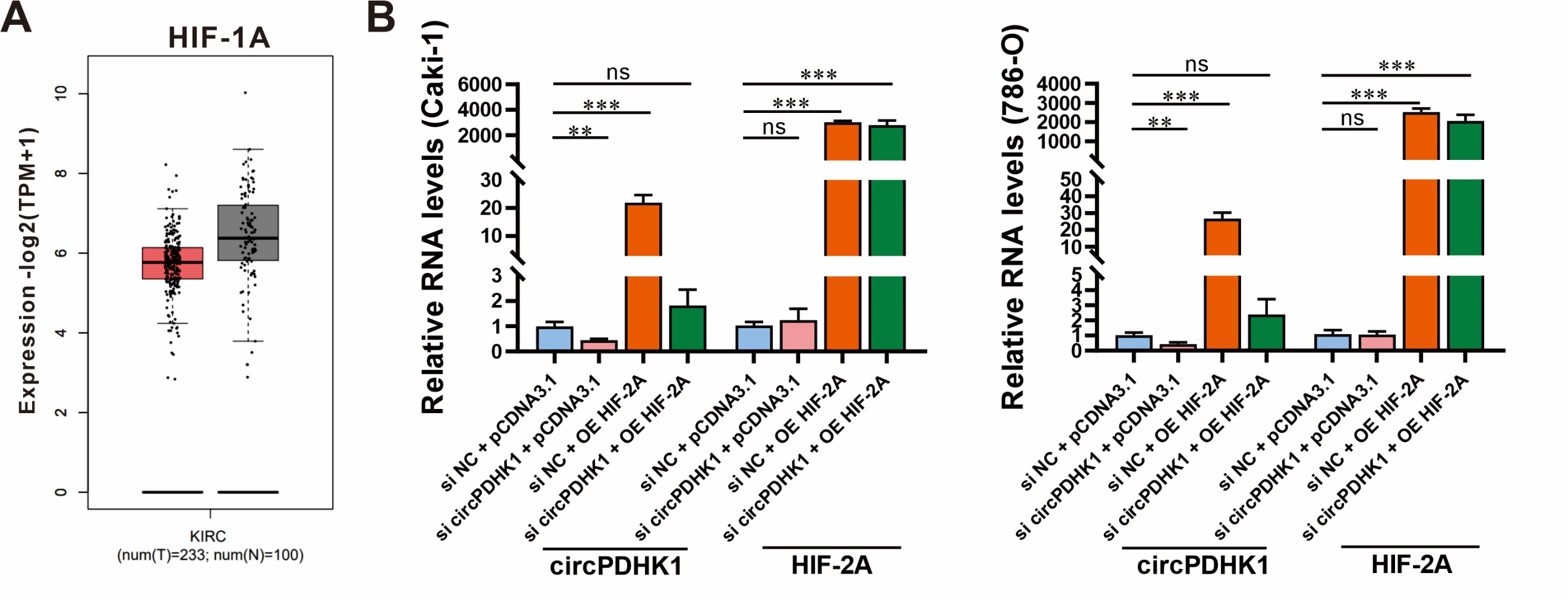


**Figure S16. Relative expression of HIF-1A in ccRCC and the expression of circPDHK1 or HIF-2A after silencing circPDHK1 or overexpressing HIF-2A in the rescue experiment. (A)** Relative expression of HIF-1A taken from data in the TCGA database (http://gepia2.cancer-pku.cn/). **(B)** RT-qPCR analysis to detected the effect of si-circPDHK1, HIF-2A overexpression, and si-circPDHK1 + OE-HIF-2A on the expression of circPDHK1 or HIF-2A in rescue experiments. ***P* < 0.01; ****P* < 0.001; ns, no significance.
